# Hypoxia-induced TGF-β–RBFOX2–ESRP1 axis regulates human MENA alternative splicing and promotes EMT in breast cancer

**DOI:** 10.1093/narcan/zcaa021

**Published:** 2020-09-18

**Authors:** Neha Ahuja, Cheemala Ashok, Subhashis Natua, Deepak Pant, Anna Cherian, Madhura R. Pandkar, Pooja Yadav, Narayanan S.S. Vishnu, Jharna Mishra, Atul Samaiya, Sanjeev Shukla

**Affiliations:** 1 Department of Biological Sciences, Indian Institute of Science Education and Research Bhopal, Madhya Pradesh 462066, India; 2 Department of Pathology, Bansal Hospital, Bhopal, Madhya Pradesh 462016, India; 3 Department of Surgical Oncology, Bansal Hospital, Bhopal, Madhya Pradesh 462016, India

## Abstract

Hypoxic microenvironment heralds epithelial–mesenchymal transition (EMT), invasion and metastasis in solid tumors. Deregulation of alternative splicing (AS) of several cancer-associated genes has been instrumental in hypoxia-induced EMT. Our study in breast cancer unveils a previously unreported mechanism underlying hypoxia-mediated AS of *hMENA,* a crucial cytoskeleton remodeler during EMT. We report that the hypoxia-driven depletion of splicing regulator ESRP1 leads to skipping of *hMENA* exon 11a producing a pro-metastatic isoform, *hMENAΔ11a.* The transcriptional repression of *ESRP1* is mediated by SLUG, which gets stimulated via hypoxia-driven TGF-β signaling. Interestingly, RBFOX2, an otherwise RNA-binding protein, is also found to transcriptionally repress *ESRP1* while interacting with SLUG. Similar to *SLUG, RBFOX2* gets upregulated under hypoxia via TGF-β signaling. Notably, we found that the exosomal delivery of TGF-β contributes to the elevation of TGF-β signaling under hypoxia. Moreover, our results show that in addition to hMENA, hypoxia-induced TGF-β signaling contributes to global changes in AS of genes associated with EMT. Overall, our findings reveal a new paradigm of hypoxia-driven AS regulation of *hMENA* and insinuate important implications in therapeutics targeting EMT.

## Introduction

Hypoxia is a hallmark of rapidly proliferating solid tumors, including breast cancer, wherein angiogenesis fails to meet the tissue growth rate, resulting in shackled oxygen diffusion to the respiring neoplastic and stromal cells (1–3). Studies reveal that the normal breast tissues exhibit a median *p*O_2_ of 65 mmHg that declines to 10 mmHg in breast cancer, the most frequent cancer among women (4,5). Hypoxic milieu leads to the dissolution of cell–cell adhesion and a dramatic reorganization of the actin cytoskeleton through epithelial–mesenchymal transition (EMT), thereby acquiring migratory and invasive phenotypes. A wealth of studies has attributed EMT to alternative splicing (AS) of a plethora of cancer-associated genes, including *CD44*, *FGFR2*, *hMENA* (also known as *ENAH*) and *p120 catenin* (6–10). Interestingly, several EMT-related AS events such as *CD44* and *CYR61* in breast cancer were shown to be governed by hypoxia (11,12). The process of EMT is associated with a remarkable reorganization of the actin cytoskeleton that causes a change in the morphology of epithelial cells with an apicobasal polarity to spindle-like mesenchymal phenotype (13,14). The hMENA, a member of the Ena/VASP family of proteins, regulates the cytoskeleton dynamics during EMT and is vastly deregulated in several cancers (15,16). Out of 15 exons of *hMENA* gene, exon 6 and exon 11a are known to be alternatively spliced in different contexts. The exon 11a containing isoform (*hMENA11a*) is reported to undermine the metastatic and invasive properties of the cells (17). The splicing switch from*hMENA11a* to*hMENAΔ11a*, wherein the 63-bp-long exon 11a is excluded, leads to a deletion of 21-amino acid region from the EVH2 domain of hMENA. The EVH2 domain, which is responsible for homo-tetramerization and contains G- and F-actin binding motifs, is phosphoregulated by EGFR signaling to alter actin filament dynamics (18,19). The phosphorylation dishevels actin polymerization at free barbed ends within the lamellipodia of cells in the course of growth factor-induced cell motility (17,19). Thus, the exclusion of exon 11a splices out the possible sites of phosphorylation and thereby supports rapid actin polymerization at the barbed ends that in turn promotes EMT. Though the hypoxic response is central to EMT, whether the AS of *hMENA* exon 11a contributes to the hypoxia-induced EMT has not been reported. Hence, here we have systematically investigated the molecular mechanism of hypoxia-induced AS of *hMENA* exon 11a and thereby its contribution to EMT in breast cancer. Our work shows that (i) hypoxia-induced TGF-β signaling contributes to *hMENA* exon 11a exclusion, (ii) hypoxia-induced exosomes contribute to the activation of TGF-β signaling, (iii) hypoxia-induced TGF-β signaling stimulates the expression of *SLUG,* downregulating *ESRP1* and thereby causing *hMENA* exon 11a exclusion, and (iv) RBFOX2 acts as a transcriptional repressor for *ESRP1,* resulting in *hMENA* exon 11a exclusion that in turn enhances EMT, and therefore paves the way for therapeutic targeting of hypoxia-driven invasion and metastasis.

## Materials and Methods

### Cell culture

Human breast cancer cell lines MCF7, HCC1806, HCC1395, MDA-MB-231, SKBR3, and T47D were obtained from American Type Culture Collection (ATCC) and were cultured in ATCC recommended media supplemented with 10% fetal bovine serum (FBS; Sigma, F7524), 100 units/ml of penicillin and streptomycin (Invitrogen, 15140122) and 2 mmol/l L-glutamine (Sigma, G7513). All cell lines were cultured in a humidified atmosphere at 37°C and 5% CO_2_. MDA-MB-231 was cultured in 0% CO_2_ at 37°C. For treatment under hypoxic conditions (1% O_2_), cells were kept in a Ruskinn INVIVO2 400 hypoxia chamber. For TGF-β treatment and inhibition, recombinant human TGF-β1 (R&D Systems, 240-B-002; 10 ng/ml) and LY-364947 (Sigma, L6293: 30 μM) were added in the media respectively.

### Breast cancer sample collection

Paraffin-embedded tumor and adjacent normal breast tissue sections (on poly-L-lysine-coated slides) were collected from Bansal Hospital, Bhopal, India. The study was approved by the Institute Ethics Committee of the Indian Institute of Science Education and Research, Bhopal, India. Informed consent was obtained from all the patients. The clinical characteristics of patients used in the study are presented in Supplementary Table S9.

### RNA interference

The MCF7 and HCC1806 cells (2 × 10^5^) were seeded in six-well culture plates. After 24 h, cells were infected with lentivirus containing small hairpin RNA (shRNA) (Sigma, Mission Human Genome shRNA Library) against ESRP1, RBFOX2, ESRP2, SLUG, ZEB1 and eGFP (shControl) with 8 μg/ml polybrene (Sigma, H9268) containing media. Cells were selected using 1 μg/ml puromycin (Sigma, P9620) for 3 days. Post-selection, cells were used for downstream experiments. For rescue experiments, overexpression of hMENA11a and hMENAΔ11a was done 2 days post-selection using Lipofectamine 2000 reagent (Invitrogen, 11668019) as per the manufacturer’s instructions. The list of shRNAs used in this study is given in Supplementary Table S8.

### CRISPR/Cas9-mediated knockout of HIF-1α

Single-guide RNA (sgRNA) targeting a constitutive exon (exon 2) of HIF-1α was designed using the online tool GPP sgRNA Designer (https://portals.broadinstitute.org/gpp/public/analysis-tools/sgrna-design). The sgRNA 5′-CTGTGATGAGGCTTACCATC-3′, targeting exon 2 of HIF-1α, was cloned into the BbsI site of the lentiviral vector pLentiCRISPR-E (Addgene, 78852). The correct cloning was verified by Sanger sequencing. The lentivirus containing sgRNA cloned plasmid was produced in HEK293T cells. HCC1806 cells were seeded in a six-well plate, and lentivirus containing medium with 8 μg/ml polybrene was used for transduction. The empty vector pLentiCRISPR-E was used as the control. Twenty-four hours post-transduction, the cells were selected using 1 μg/ml puromycin for 2–5 days. The selected cells were used to generate single-cell suspension in a 96-well plate by a serial dilution method. The single cell-derived clones were screened for the knockout by western blot and by Sanger sequencing of the region targeted by sgRNA.

### Microarray expression profiling (Human Transcriptome Array 2.0)

MCF7 and HCC1806 cells were cultured in 1% O_2_ (hypoxic) and 21% O_2_ (normoxic) for microarray profiling of AS under hypoxia. For profiling of AS under TGF-β induction, MCF7 and HCC1806 cells were treated with TGF-β ligand (10 ng/ml). All samples were lysed using TRI-zol reagent (Invitrogen), and total RNA was isolated using the PureLink RNA Mini Kit (Invitrogen). One hundred nanograms of total RNA was used to prepare biotinylated cDNA using GeneChip™ WT Plus Reagent Kit (Applied Biosystems) as per the instructions given by the manufacturer. After amplification of cDNA, fragmentation was done, and 5.5 μg of fragmented cDNA was hybridized on Affymetrix GeneChip™ Human Transcriptome Array 2.0 (HTA 2.0) chips at 45°C for 16 h. The chips were then washed and stained in the Affymetrix Fluidics Station 450. After hybridization, the fluorescence intensity of the arrays was scanned using the Affymetrix Scanner 7G. The raw CEL files generated after the scan were used for further analysis.

### Human Transcriptome Array 2.0 data analysis

CEL files generated after scanning were analyzed for differential AS on Transcriptome Analysis Console (TAC) 4.0 using the gene+exon–SST-RMA method of summarization. The results of the microarray analysis were deposited in Gene Expression Omnibus (GEO) under accession number GSE147516. Splicing events with absolute splicing index (|SI|) ≥1.5 and *P* < 0.05 were considered to be significant. The significant events were represented by a scatter plot made using ggplot2 package in R. A heat map was constructed for top 50 cassette exon exclusion and inclusion events under hypoxia using the online tool Morpheus (https://software.broadinstitute.org/morpheus). For finding events pertaining to EMT, the top 10 000 inclusion and top 10 000 exclusion events were taken into consideration. Out of these top events, those corresponding to genes that are known to get spliced during EMT (20) were shortlisted. The list of genes that get differentially spliced during EMT (|ΔPSI| ≥ 0.1 and *q*-value <0.05) were obtained from the RNA sequencing (RNA-seq) data by Hu *et al.* (20) (GSE139074). These genes were shown to have differential splicing in a tamoxifen-inducible HMLE/Twist-ER model of EMT (Supplementary Table S4). Eventually, our search was narrowed down to genes that are involved in the regulation of the actin cytoskeleton (genes contained in the gene signature KEGG_REGULATION_OF_ACTIN_CYTOSKELETON were selected). A final list of events belonging to 16 genes for MCF7 and 10 genes for HCC1806 was obtained (Supplementary Table S1). Venn diagrams were constructed to find the overlap between the genes that undergo exon inclusion or exclusion under hypoxia, TGF-β treatment and during EMT using Venn Diagram Plotter (https://omics.pnl.gov/software/venn-diagram-plotter) (21) (Supplementary Table S3). Over-representation analyses (ORAs) to obtain significantly enriched Gene Ontology (GO) terms in the set of genes that showed differential exon inclusion or exclusion in all three conditions were performed using the Web-Based Gene Set Analysis Toolkit (WebGestalt) (22). False discovery rate (FDR)-corrected *P*-value of <0.05 was considered significant while obtaining enriched GO terms. For HTA 2.0 triple-negative breast cancer patient profile (GSE76250) (23), CEL files for 165 tumor samples were downloaded from GEO, and the gene expression levels were obtained using oligo package in R (24). The HYPOXIA_HALLMARK signature from Molecular Signature Database (25) was used to stratify the patient samples into hypoxic or normoxic using a similar strategy as described previously (26). The sum of expression levels corresponding to genes contained in the HYPOXIA_HALLMARK signature was calculated (hypoxia activation score) for all the samples. The samples having higher hypoxia activation scores (top 20%) were considered as hypoxic, whereas samples with lower scores (bottom 20%) were considered as normoxic. Raw CEL files for these stratified samples were then used to perform differential AS analysis using TAC 4.0. Details of all gene signatures used are provided in Supplementary Table S5.

### Analysis of The Cancer Genome Atlas data

The Cancer Genome Atlas (TCGA) RNA-seq gene expression data for invasive breast carcinoma samples were downloaded from https://xenabrowser.net/. The dataset consisted of 1099 tumor and 113 normal samples. To check the enrichment of hypoxia pathway genes (using the WINTER_HYPOXIA_UP gene signature) in normal versus tumor samples, gene set enrichment analysis was performed using GSEA software (27). Stratification of tumor samples was then done, as described previously (26). Hypoxia activation score was calculated by summing up the expression of genes contained in the WINTER_HYPOXIA_UP signature for each tumor sample, and then the samples were ranked on the basis of this score. Those with higher scores (top 20%) were categorized as Hypoxic_high and those with lower scores (bottom 20%) as Hypoxic_low GSEA for various signaling pathways (Supplementary Table S2) and EMT between these stratified samples was then performed. Details of all gene signatures used in GSEA or for stratification are provided in Supplementary Table S5. To check the confidence of stratification, unsupervised *K-mea*ns clustering was done and clusters were then visualized using a scatter plot after principal component analysis.

### RBFOX2 ChIP-seq analysis

Chromatin immunoprecipitation sequencing (ChIP-seq) data corresponding to RBFOX2 were downloaded from GEO (GSE106042). The transcription start sites (TSSs) of all annotated human genes were downloaded from Biomart by Ensembl. Bedtools toolset was used to compare the genomic positions of the ChIP-seq peaks relative to TSSs of all genes. A region of 2 kb around the TSS was considered to be the promoter region. ChIPseeker package in R (28) was used for generating occupancy profile and distribution of peaks as well as for obtaining enriched pathways among genes with RBFOX2 binding on promoter.

### Molecular cloning

The *ESRP1, hMENA11a* and *hMENAΔ11a* were cloned in pCMV-3Tag1a (Agilent, 240195) overexpression plasmid from MCF7 cDNA using Dream-Taq DNA Polymerase (Thermo Fisher Scientific, EP0702). Primers were as follows: ESRP1 Fw (5′-CGGGATCCATGACGGCCTCTCCGGATTA-3′), ESRP1 Rev (5′-CCCAAGCTTTTAAATACAAACCCA TTCTTTGGG-3′), hMENA11a/hMENAΔ11a Fw (5′-CGGAATTCATGAGTGAACAGAGTATCTGTCAGG-3′) and hMENA11a/hMENA11Δ Rev (5′-ACGCGT CGACCTATGCAGTATTTGACTTGCTCAGTT-3′). ESRP1 was cloned between the BamHI F and HindIII R sites and hMENA11a/hMENA11Δ was cloned between the EcoRI F and Sa1I R sites.

### Chromatin immunoprecipitation

The binding of transcription factors (TFs) on target promoters was evaluated by performing the ChIP assay, as described previously (29). Briefly, lysed cells were sonicated (approximate chromatin fragment length: 200–500 bp), and chromatins (25 μg) were immunoprecipitated with concomitant antibody followed by overnight incubation at 4° C. The following antibodies were used for ChIP: anti-human pSMAD2/3 (S465/S467) (R&D Systems, MAB8935, lot no. CJRA0318051), anti-SLUG (Sigma, PRS3959, lot no. 87711801), anti-RBFOX2 (Abcam, ab57154, batch no. GR317972-9), normal rabbit IgG (Millipore, 12-370, lot no. 2295402) and normal mouse IgG (Millipore, 12-371B, lot no. 2332526). The immunoprecipitated protein–DNA complexes and 5% input were analyzed by qRT-PCR in triplicate using specific primers (Supplementary Table S10) flanking the predicted binding sites and SYBR Green Master Mix (Promega, A6002, lot no. 0000385100). All the ChIP experiments were performed at least thrice. IP values were normalized to input using the following formula: 2^^^(Ct_input – Ct_immunoprecipitation) (30). Resultant values were subsequently normalized to IgG control IP values. The significance between two different groups was identified using Student’s *t*-test. *P* < 0.05 was considered statistically significant.

### Real-time PCR

qRT-PCR was done as previously described (30). Total RNA was extracted from breast cancer cells using the TRI-zol (Invitrogen, 15596026) according to the manufacturer’s instructions. The RNA concentrations were measured using NanoDrop (Thermo Fisher Scientific, ND8000). cDNA was synthesized from 1 μg of total RNA by PrimeScript 1st strand cDNA Synthesis Kit (TaKaRa, 6110A, lot no. AJX1015N) as per the manufacturer’s instructions. Amplification reactions were performed on light cycler 480 II (Roche) using SYBR Green (Affymetrix, 75665) according to the manufacturer’s instructions. Each reaction was carried out in triplicate. The average cycle thresholds from biological triplicates were calculated and normalized to control gene RPS16 using the following formula: 2^^^(Ct_control – Ct_target). Also, to control gene normalization, exon-level expressions were normalized to a constitutive exon. The Student’s *t*-test was performed to compare gene/exon expression between two distinct groups. *P* < 0.05 was considered as statistically significant.

### Semi-quantitative PCR

Total RNA was extracted from breast cancer cells using the TRIzol (Invitrogen, 15596026) according to the manufacturer’s instructions. The RNA concentrations were measured using NanoDrop (Thermo Fisher Scientific, ND8000). cDNA was synthesized from 1 μg of total RNA by PrimeScript 1st strand cDNA Synthesis Kit (TaKaRa, 6110A, lot no. AJX1015N) as per the manufacturer’s instructions. cDNA was amplified using Dream Taq polymerase (Thermo Fisher Scientific, EP0702) in PCR reactions consisting of 35 cycles using flanking primers as given in Supplementary Table S7. PCR products were analyzed on a 2% agarose gel after ethidium bromide staining.

### Co-immunoprecipitation

The SLUG and RBFOX2 interactions were evaluated by performing co-immunoprecipitation (co-IP), as described previously (31). Briefly, the hypoxia-treated MCF7 cells were lysed using a lysis buffer [10 mM Tris (pH 7.5), 150 mM NaCl, 0.5 mM EDTA, 0.5% NP-40 and protease inhibitor cocktail (PIC; Roche)]. The lysates were incubated with anti-SLUG (Sigma, PRS3959, lot no. 87711801) or anti-RBFOX2 (Abcam, ab57154, batch no. GR317972-9) antibody along with normal rabbit IgG (Millipore, 12-370, lot no. 2295402) or normal mouse IgG (Millipore, 12-371B, lot no. 2332526) for 2 h at 4°C. Subsequently, 25 μl Protein-G Dynabeads (Thermo Fisher Scientific, 10004D, lot 0078227) were added to the immunoprecipitated lysate and further incubated for 8 h at 4°C. The beads were then washed thrice with the lysis buffer and boiled with 2× Laemmli buffer for 5 min. Immunoblotting analysis was performed with the eluted proteins with anti-SLUG and anti-RBFOX2 antibodies.

### Generation of promoter deletion constructs

To generate promoter constructs, human ESRP1 (gene ID: 54845) promoter sequence was retrieved from the Eukaryotic Promoter Database (https://epd.vital-it.ch/). The ESRP1 gene promoter from −1748 bp upstream to +110 bp downstream of the TSS at +1 was amplified by PCR and inserted in the pGL3-Basic expression vector (Promega) using genomic DNA as a template, and the deletion construct ESRP1-1482 was derived from pGL3-1748. Primers were as follows: ESRP1-1748 Fw (5′- GGGGTACCCTGGCCTT CGCCCGCTCTCA-3′), ESRP1–1482 Fw (5′-GGGGTA CCGGCTGGACACCTAGAGCCGA-3′) and Rev (5′-CG GCTAGCAGGCGGTAAGGTGGTGTGGA-3′). ESRP1 deletion construct was cloned between the KpnI F and NheI R sites.

### Luciferase reporter assays

MCF7 cells (0.05 × 10^6^) were seeded in 24-well plates and cultured for 16 h. The cells were co-transfected with different ESRP1 promoter–luciferase constructs, and pRL-TK Renilla luciferase plasmid (Promega, E2231) harvested at 48 h and lysed in passive lysis buffer. For TGF-β and normoxic/hypoxic experiments, cells were transfected with SBE4-Luc (Addgene, 16495) or pBV-Luc (Addgene, 16539). After 12 h of transfection, respective treatments were given. The firefly luciferase activities were measured in a GloMax-Multi Detection System (Promega), and the values were normalized to Renilla luciferase activities. The relative values are represented as mean ± SD of triplicates from a representative experiment.

### Site-directed mutagenesis

The site-directed mutant construct of the ESRP1 promoter was prepared using oligonucleotides with mutations in the RBFOX2 binding site (TGCATG). The SDM primers were as follows: Fw (5′-TCTGCAGGGATAGG TaaATaGTTGCCCGTTTCAC-3′) and Rev (5′-GTGA AACGGGCAACtATttACCTATCCCTGCAGA-3′). The mutated nucleotides are represented by lowercase letters and underlined. The ESRP1 promoterSDM was confirmed by DNA sequencing after the digestion of non-mutated vectors with the endonuclease DpnI (TaKaRa, 1235A).

### Immunoblotting

The cells were lysed using urea lysis buffer (8 M urea, 2 M thiourea, 2% CHAPS, 1% DTT) and 1× PIC (leupeptin 10–100 μM, pepstatin 1 μM, 1–10 mM EDTA, <1 mM AEBSF), spun at maximum speed (16 900 × g) in a 4°C centrifuge. The supernatant was separated, quantified and equal concentration of protein samples was loaded. Quantification of the bands was done using ImageJ software. Details of antibodies used for immunoblotting are provided in Supplementary Table S6.

### Phalloidin assay

After puromycin selection, cells were transfected with overexpression plasmids pCMV-hMENA11a and pCMV-hMENAΔ 11a. Cells were seeded in 12-well plates on coverslips. After 48 h of hypoxic or normoxic treatment, cells were washed and fixed using 4% formaldehyde and permeabilized using 0.1% Triton X-100. After washing, cells were then incubated with FITC-conjugated phalloidin (Sigma, P5282) for 4 h, washed with phosphate-buffered saline (PBS) and mounted. Sections were analyzed using an Olympus FV3000 confocal laser scanning microscope with a 60× Plan Apo N objective (oil, 1.42 NA).

### RNA immunoprecipitation

The RNA IP assay was performed as described previously (32). Briefly, after 24 h of hypoxic/normoxic treatment, 10–20 million cellswere washed with PBS and lysed with 4 ml of ice-cold swelling buffer A (25 mM HEPES, 1.5 mM MgCl_2_, 85 mM KCl, pH 8.0) for 5 min. Cells were scraped and centrifuged at 1350 × *g* for 5 min at 4°C, the pellet was lysed in 1 ml of buffer C (25 mM HEPES, 1.5 mM MgCl_2_, 85 mM KCl, pH 8.0, 0.2% NP-40, 1% Triton X-100, 1× PIC, 2 U/ml of RNaseOUT, 5 mM sodium fluoride, 5 mM sodium orthovanadate, 5 mM β-glycerophosphate) for 30 min and cleared by centrifugation at 21 000 × *g* for 10 min. The supernatant was split into two fractions of 500 μl each for IgG and IP. Five percent aliquot of the supernatant was incubated as input. Specific antibodies (details of antibodies are given in Supplementary Table S6) were added to the IP supernatant and incubated for 1–2 h at 4°C with gentle rotation. Forty microliters of Dynabeads was added to each sample and incubated for 1 h at 4°C with gentle rotation. Magnetic rack was used to remove the supernatant and beads were washed three times with 500 μl of buffer D (25 mM HEPES, 1.5 mM MgCl_2_, 85 mM KCl, pH 8.0, 0.02% NP-40, 0.25% Triton X-100, 1× PIC, 0.1 U/ml of RNase-OUT, 5 mM sodium fluoride, 5 mM sodium orthovanadate, 5 mM β-glycerophosphate). Beads were resuspended in TRIzol RNA extraction reagent (Invitrogen, 15596026), and RNA was isolated according to the manufacturer’s instructions. cDNA was synthesized using total RNA isolated by PrimeScript 1st strand cDNA Synthesis Kit (TaKaRa, 6110A, lot no. AJX1015N) as per the manufacturer’s instructions. Primers used for downstream real-time PCR are provided in Supplementary Table S7 for exon 3 of hMENA.

### Exosome isolation

The cells were cultured in exosome-depleted media for all the exosome-related experiments. To remove the residual bovine exosomes from the FBS, Dulbecco’s modified Eagle medium and RPMI 1640 medium containing 20% FBS were centrifuged overnight at 100 000 × *g* at 4°C The FBS concentration was made up to 10% by adding serum-free media. For exosome isolation from conditioned media, the ExoEnrich™ (ExoCAN, PEC-25, lot no. 1220161) kit was used as per the manufacturer’s instructions. Dynamic light scattering (Delsa™ Nano C, Beckman Coulter) was used to obtain the size distribution of isolated exosomes. The isolated exosomes were lysed using the ExoLyseP (ExoCAN, PEL-25P, lot no. 1220161) kit.

### Immunohistochemistry

The study was approved by the Institute Ethics Committee of the Indian Institute of Science Education and Research Bhopal, India. Informed consent was obtained from all the patients. Formalin-fixed, paraffin-embedded human breast cancer tissue sections were obtained from Bansal Hospital, Bhopal, India. Immunohistochemistry was performed according to the experimental protocol of the Vectastain ABC elite kit (Vector Laboratories, Burlingame, CA, USA) (33), visualized with the DAB (3,3′-diaminobenzidine, Sigma) chromogenic method and counterstained with Harris’ hematoxylin (Merck). Slides were fixed overnight at 65°C in the water bath, deparaffinized and rehydrated as per the standard procedure. Subsequently, 10 mM sodium citrate buffer (pH 6)-based antigen retrieval was done in the laboratory microwave for 10 min. Endogenous peroxidase was quenched with 1:10 dilution of 3% hydrogen peroxidase in methanol, followed by blocking with 3% bovine serum albumin (BSA). Primary antibodies against CAIX (1:50), phospho-SMAD2/3 (1:50), hMENA11a (1:200) and ESRP1 (1:50) were used (details of the antibodies are provided in Supplementary Table S6). Sections were examined using the Thermo Scientific™ Invitrogen™ EVOS™ FL Auto 2 Imaging System and at 40× magnification. Images were then processed in Adobe Photoshop CS Version 8.0. Quantification was done using color deconvolution in ImageJ (Fiji) and mean gray values were then converted to optical density.

### Invasion assay

Invasion assays were performed as described previously (34). The puromycin-selected 2 × 10^4^ cells were added to the upper chamber of transwell (Corning) over Matrigel (Corning) layer and incubated for 48 h in a cell culture incubator or a hypoxia chamber. The non-migrated cells in the upper layer of Matrigel were removed, and cells migrated to the lower chamber of transwell were fixed in 4% formaldehyde and then stained with 0.05% crystal violet in 10% methanol. Five random fields were counted using an inverted microscope (Olympus CKX41).

### Immunofluorescence

Cells (3 × 10^4^) were seeded in 12-well plates on coverslips. After TGF-β and hypoxic/normoxic treatments, cells were washed with PBS, fixed using 4% formaldehyde and permeabilized using 0.1% Triton X-100. Cells were then blocked using 2% BSA for 1 h at room temperature followed by primary antibody phospho-SMAD2 (Ser465/467)/SMAD3 (Ser423/425) (CST, 8828S, lot no.7) incubation overnight in 4◦C. Cells were then washed with PBS and incubated with Alexa-Flour 555 anti-rabbit IgG (Invitrogen, A32732, lot no. 1858260) for 1 h at room temperature and counter-stained with Hoechst. Coverslips were then mounted and analyzed by Thermo Scientific™ Invitrogen™ EVOS™ FL Auto 2 Imaging System.

## Results

### Hypoxia promotes exon 11a exclusion from *hMENA* pre-mRNA in breast cancer cells

**Figure 1:**
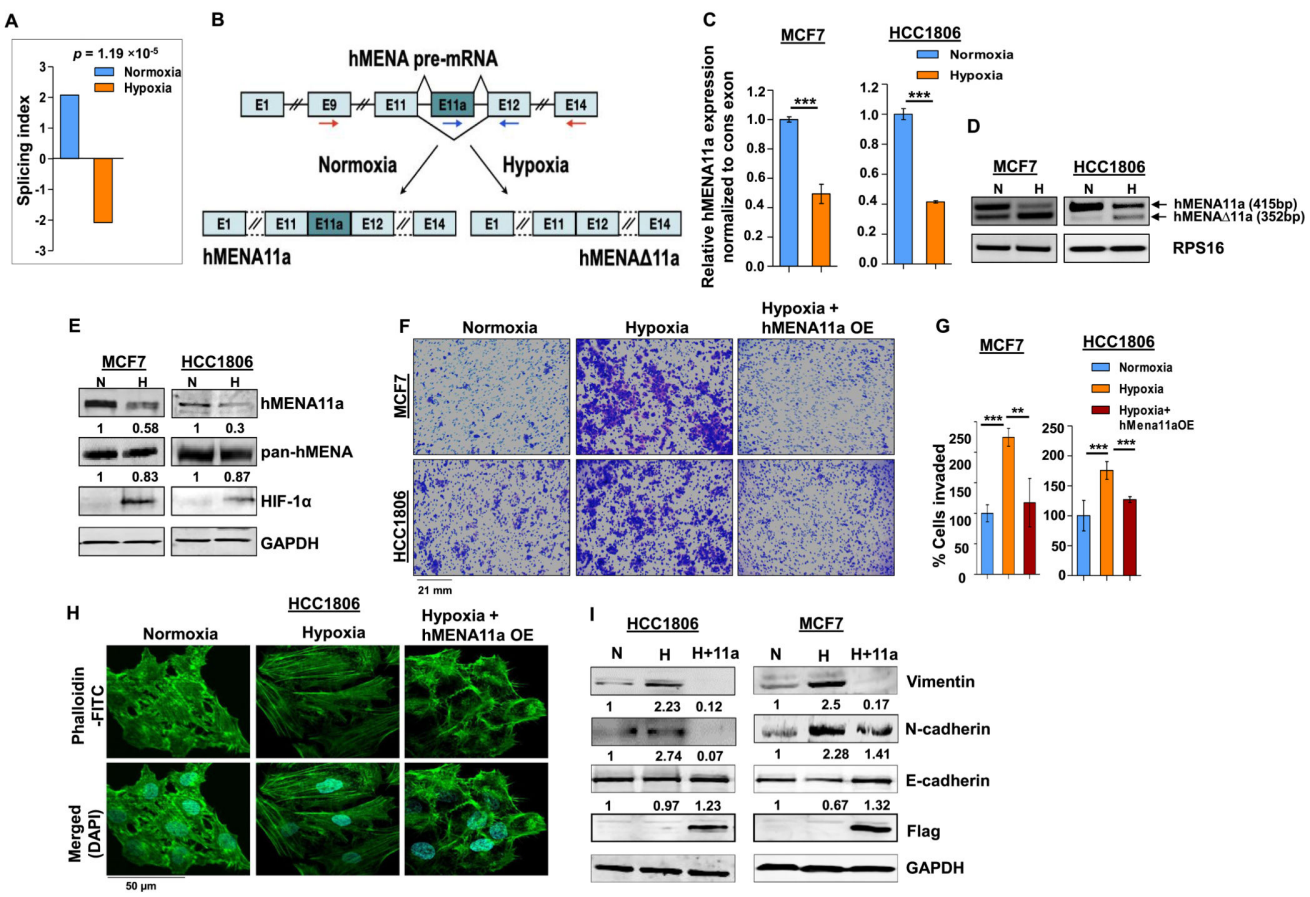
hMENA11a isoform is downregulated under hypoxia, augmenting actin polymerization and invasive properties of breast cancer cells. (**A**) Triple-negative breast cancer samples (*n* = 165) from the microarray dataset GSE76250 were stratified into normoxic and hypoxic, followed by AS analysis to check the levels of inclusion of exon 11a of *hMENA* gene. The reversal of the inclusion of exon 11a in hypoxic samples as compared to normoxic samples is represented by the SI values. (**B**) Schematic of*hMENA* splicing demonstrating the exclusion and inclusion of exon 11a in hypoxia and normoxia, respectively. Red arrows indicate position of primers used for semi-quantitative PCR and blue arrows indicate positions of qRT-PCR primers. (**C**) Real-time PCR for *hMENA* 11a isoform (Ct values normalized to RPS16) and (**D**) semi-quantitative PCR of *hMENA* (using exon9 forward and exon15 reverse primers) showing upper (exon 11a included) and lower (exon 11a excluded) bands (RPS16 used as a control), 24 h after hypoxic treatment in MCF7 and HCC1806. (**E**) Immunoblotting of hMENA11a, pan-hMENA and HIF-1α under normoxia (N) and hypoxia (H) in MCF7 and HCC1806 (GAPDH as a control). (**F**) Invasion assay and (**G**) its quantification (as % cells invaded), in MCF7 and HCC1806, and (**H**) phalloidin staining, in HCC1806, after 48 h under normoxia, hypoxia and hMENA11a overexpression (OE) under hypoxic conditions. (**I**) Immunoblotting of vimentin, N-cadherin, E-cadherin and Flag under normoxia, hypoxia and hMENA11a overexpression in HCC1806 and MCF7 (GAPDH as a control). Error bars show mean values ± SD (*n* = 3 unless otherwise specified). As calculated using two-tailed Student’s *t*-test, **P < 0.01 and ***P < 0.001.

Cancer cells, while adapting to hypoxic stress, instigate a more aggressive phenotype by rewiring gene expression programs (35). However, the role of AS events in breast cancer cells that occur during low-oxygen stress leading to oncogenesis has remained largely obscure. To identify hypoxia-elicited AS events, we performed HTA 2.0 microarray in breast cancer cell lines MCF7 and HCC1806 under normoxic (21% O_2_) or hypoxic (1% O_2_) conditions. Among several actin cytoskeleton remodeling proteins (Supplementary Table S1) that showed noteworthy splicing switch under hypoxia, *hMENA* was selected on the strength of its well-documented role in EMT (19). Although AS of *hMENA* is reported to be associated with EMT, the role of hypoxia in driving AS of *hMENA* remained largely unexplored. The HTA 2.0 array data from MCF7 and HCC1806 cell lines show a significant exclusion *(P* = 4.22 × 10^−5^ and 0.0026, respectively) of exon 11a from *hMENA* pre-mRNA under hypoxia (Supplementary Figure S1A). To validate the cell line data, we stratified 165 tumor samples from a breast cancer patient microarray profile (GSE76250) (23) as hypoxic or normoxic based on the HYPOXIA_HALLMARK (25) gene signature using a method described previously (26). It appeared that the exon 11a is significantly excluded *(P* = 1.19 × 10^−5^) in samples stratified as hypoxic as compared to those stratified as normoxic (Figure 1A). The real-time PCR using specific primers (Figure 1B and C) and semiquantitative PCR (Figure 1D and Supplementary Figure S1B) using the flanking primers (arrows shown in Figure 1B) also show a significant decrease in the expression of the *hMENA11a* isoform under hypoxia in various breast cancer cell lines. Consistent with the role of hMENAΔ11a in invasion (19), MDA-MB-231, a highly invasive cell line, shows an ablated expression of the *hMENA11a* isoform (Supplementary Figure S1B). The same phenomenon is implicated in the immunoblot with the antibody exclusively targeting hMENA11a isoform (Figure 1E and Supplementary Figure S1C). Additionally, *hMENA* exon 11a AS appears to be HIF-1α dependent as the CRISPR/Cas9-mediated knockout of HIF-1α resulted in an upregulation of hMENA11a (Supplementary Figure S1F and G). The AS of the *hMENA* pre-mRNA generates another cancer-specific splice isoform lacking exon 6 (19). Nevertheless, splicing of *hMENA* exon 6 is not affected by hypoxia as the expression of *hMENAv6* isoform is invariant in both the conditions (Supplementary Figure S1D). These results indicate that the expression of the epithelial-specific isoform *of hMENA* (i.e. *hMENA11a)* significantly declines under hypoxia as compared to normoxia, highlighting the role of hMENAΔ11a in hypoxia-induced EMT. Further, to explore the role of hypoxia in breast cancer cell invasion, we performed the transwell invasion assay for MCF7 and HCC1806 cells. We found that hypoxic cells exhibited augmented invasion as compared to normoxic cells (Figure 1F and G). Since actin cytoskeleton remodeling and stress fiber formation are hallmarks of EMT (36), we have performed phalloidin staining in MCF7 and HCC1806 cells to verify the role of hypoxia in cytoskeleton remodeling. Normoxic cells showed a largely cortical organization of phalloidin-labeled F-actin with a cobblestone morphology. On the contrary, 48-h hypoxia treatment to cells resulted in a mesenchymal-like elongated phenotype with the reorganization of the actin cytoskeleton into aggregated actin stress fibers (Figure 1H and Supplementary Figure S1E).

Further, to investigate whether the hypoxia-driven alteration of phenotype and invasive behavior of breast cancer cells is reliant on decreased *hMENA11a* expression, we ectopically expressed the *hMENA11a* isoform in MCF7 and HCC1806 cells under hypoxia, and performed invasion and phalloidin staining assays. We observe a significant reduction in the invasiveness (Figure 1F and G) and actin stress fiber formation (Figure 1H and Supplementary Figure S1E) upon *hMENA11a* overexpression under hypoxia. In accordance with this observation, the expression of the mesenchymal markers such as N-cadherin and vimentin declined substantially, along with an elevation in E-cadherin level when hypoxic cells were overexpressed with *hMENA11a* (Figure 1I). These results indicate that the diminishment of *hMENA11a* expression is critical for hypoxia-driven EMT.

### Hypoxia-induced TGF-β signaling results in exon 11a exclusion

To investigate the mechanism of hypoxia-induced AS of *hMENA* exon 11a, we performed GSEA using TCGA RNA-seq data consisting of 1099 tumor and 113 normal samples. It emerged that the hypoxia-associated genes were significantly enriched in the tumor set (Supplementary Figure S2A). Next, we stratified the tumor samples into two groups (Hypoxic_high and Hypoxic_low) based on the degree of hypoxia activation using a method described previously (26) (Supplementary Figure S2B), followed by GSEA to check for the enrichment of various signaling pathways implicated in EMT (37) (Supplementary Table S2). Notably, expression of the TGF-β signaling pathway genes was found to be significantly enriched in tumor samples with a higher degree of hypoxia activation *(n =* 220) against those with a lower degree of hypoxia activation *(n =* 220; Supplementary Figure S2C). Further analysis with these stratified tumor samples revealed a positive association of EMT-related genes with hypoxia (Supplementary Figure S2D), suggesting a possible involvement of TGF-β signaling in the acquisition of EMT under hypoxia. Our *in silico* analysis is in agreement with the prior studies correlating hypoxia with EMT (38), hypoxia with TGF-β (39) and TGF-β with EMT individually (40). Therefore, we hypothesize that the hypoxia-induced hMENA exon 11a AS and thereby EMT is under the control of TGF-β signaling.

**Figure 2:**
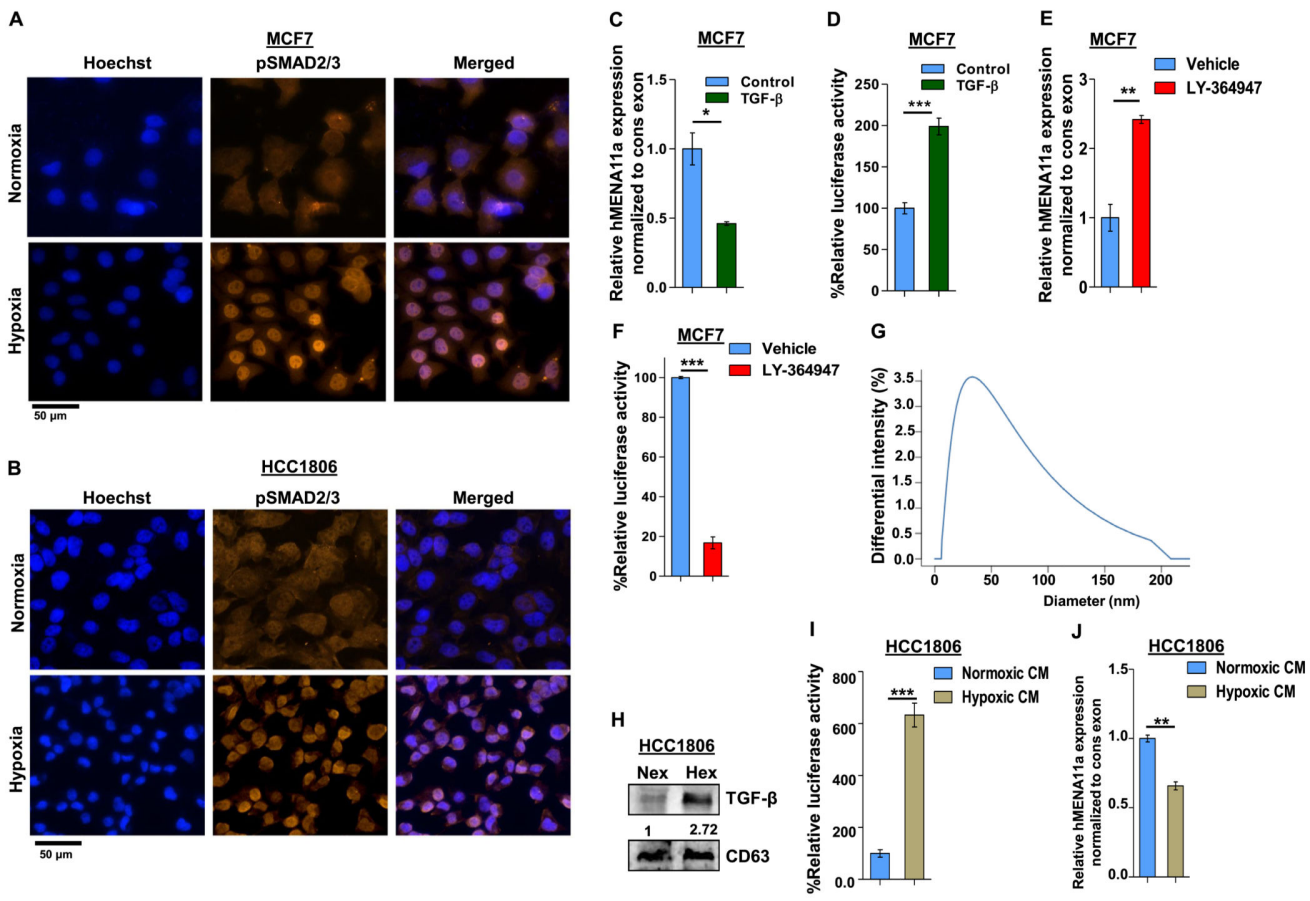
Activation of TGF-β signaling under hypoxia causes exon 11a exclusion from *hMENA* pre-mRNA. Immunofluorescence of pSMAD2/3 showing enhanced nuclear localization under hypoxia as compared to normoxia in MCF7 (**A**) and HCC1806 (**B**) after 12 h. (**C**) qRT-PCR for *hMENA11a* isoform 24 h after TGF-β induction in MCF7 (Ct values normalized to RPS16). (**D**) Relative luciferase activity (SBE4:Luc) after 12 h of TGF-β (10 ng/ml) induction in MCF7 versus control. (**E**) qRT-PCR for *hMENA11a* after 24 h of TGF-β signaling inhibition under hypoxia in MCF7. (**F**) Relative luciferase activity (SBE4:Luc) after 12 h of TGF-β signaling inhibition in MCF7 using 30 μM LY-364947 inhibitor under hypoxia. (**G**) Dynamic light scattering analysis (done using conditioned media of hypoxia-treated HCC1806) showing particles with a size distribution of 20–200 nm. Most of the particles fall in the range defined for exosomes (40–100 nm). (**H**) Immunoblot for TGF-β in normoxic and hypoxic exosomes (CD63 exosomal marker was used as a control) collected from media of HCC1806 treated under normoxia and hypoxia, respectively. (**I**) Relative luciferase activity (SBE4:Luc) after 12 h of normoxia- and hypoxia-conditioned (CM) media treatment in HCC1806. (**J**) qRT-PCR for *hMENA11a,* 24 h after normoxia- and hypoxia-conditioned treatment in HCC1806. Error bars show mean values ± SD (*n =* 3 unless otherwise specified). As calculated using two-tailed Student’s *t* -test, *P < 0.05, **P < 0.01 and ***P < 0.001.

Upon ligand binding, the TGF-β receptors get phosphorylated and further activate SMAD2/3 by phosphorylation (41–43). We examined the upregulation of TGF-β signaling in cells under hypoxia by using the SMAD binding element 4:luciferase promoter construct (SBE4:Luc) containing four copies of the SMAD binding site upstream of a luciferase reporter gene. The luciferase activity increased drastically after 12-h hypoxia treatment in both MCF7 and HCC1806 cells (Supplementary Figure S2E). Similarly, immunofluorescence experiment with anti-pSMAD2/3 antibody also reflects elevated TGF-β signaling after 12-h hypoxia treatment to MCF7 and HCC1806 cells (Figure 2A and B). Hence, to confirm the entanglement of hypoxia-induced TGF-β signaling in *hMENA* AS, we stimulated the MCF7 cells with TGF-β ligand (10 ng/ml) under normoxia. This led to the exclusion *of hMENA* exon 11a as confirmed by real-time PCR and immunoblotting (Figure 2C and Supplementary Figure S2G). Activation of TGF-β signaling upon TGF-β ligand treatment is confirmed by the luciferase assay (Figure 2D) as well as by immunofluorescence (Supplementary Figure S2F). For further validation of the role of TGF-β signaling in the exon 11a exclusion, we treated both the cell lines with LY-364947 (30 μM), which is a potent and selective ATP-competitive inhibitor of TGF-β receptor kinase I, under hypoxia (44). The inhibitor treatment reverted the hMENA11a exclusion event under hypoxia, as shown by real-time PCR (Figure 2E) and immunoblotting (Supplementary Figure S2H). The inhibition of TGF-β signaling after LY-364947 treatment is verified by the luciferase assay (Figure 2F). We also observed that the invasiveness and actin stress fiber formation are augmented in breast cancer cells when treated with TGF-β ligand (Supplementary Figure S2I–K). Next, we wanted to investigate how hypoxic stress contributes to TGF-β signaling activation. Wang *et al.* showed that the hypoxic breast cancer cells tend to augment exosome production in an HIF-dependent manner, wherein the HIF-driven expression of the small GTPase RAB22A is critical for microvesicle formation (45). We verified the same with immunoblot analysis using exosomal marker CD63 after isolating exosomes from cell culture supernatant for both normoxic and hypoxic conditions (Supplementary Figure S2L). The size distribution profile, as obtained from dynamic light scattering, confirmed the exosomal nature of extracellular vesicles isolated from conditioned media (Figure 2G). Interestingly, it was observed that the hypoxic cells package a greater amount of TGF-β in secreted exosomes as compared to normoxic cells (Figure 2H and Supplementary Figure S2M). Next, we treated HCC1806 and MCF7 cells containing SBE4:Luc promoter construct with hypoxia-conditioned culture media and observed a steep increase in the luciferase activity (Figure 2I and Supplementary Figure S2N). The treatment with hypoxia-conditioned media also caused significant *hMENA* exon 11a skipping in normoxic HCC1806 (Figure 2J) and MCF7 cells (Supplementary Figure S2O). Moreover, hypoxia-conditioned media treatment enhanced actin stress fiber formation in HCC1806 cells, as shown by the phalloidin assay (Supplementary Figure S2P). These results unravel the novel role of hypoxia-induced exosomes in AS *of hMENA* 11a mediated by TGF-β signaling.

### The decrease in the ratio of ESRP1 to RBFOX2 causes exclusion of *hMENA* exon 11a

**Figure 3:**
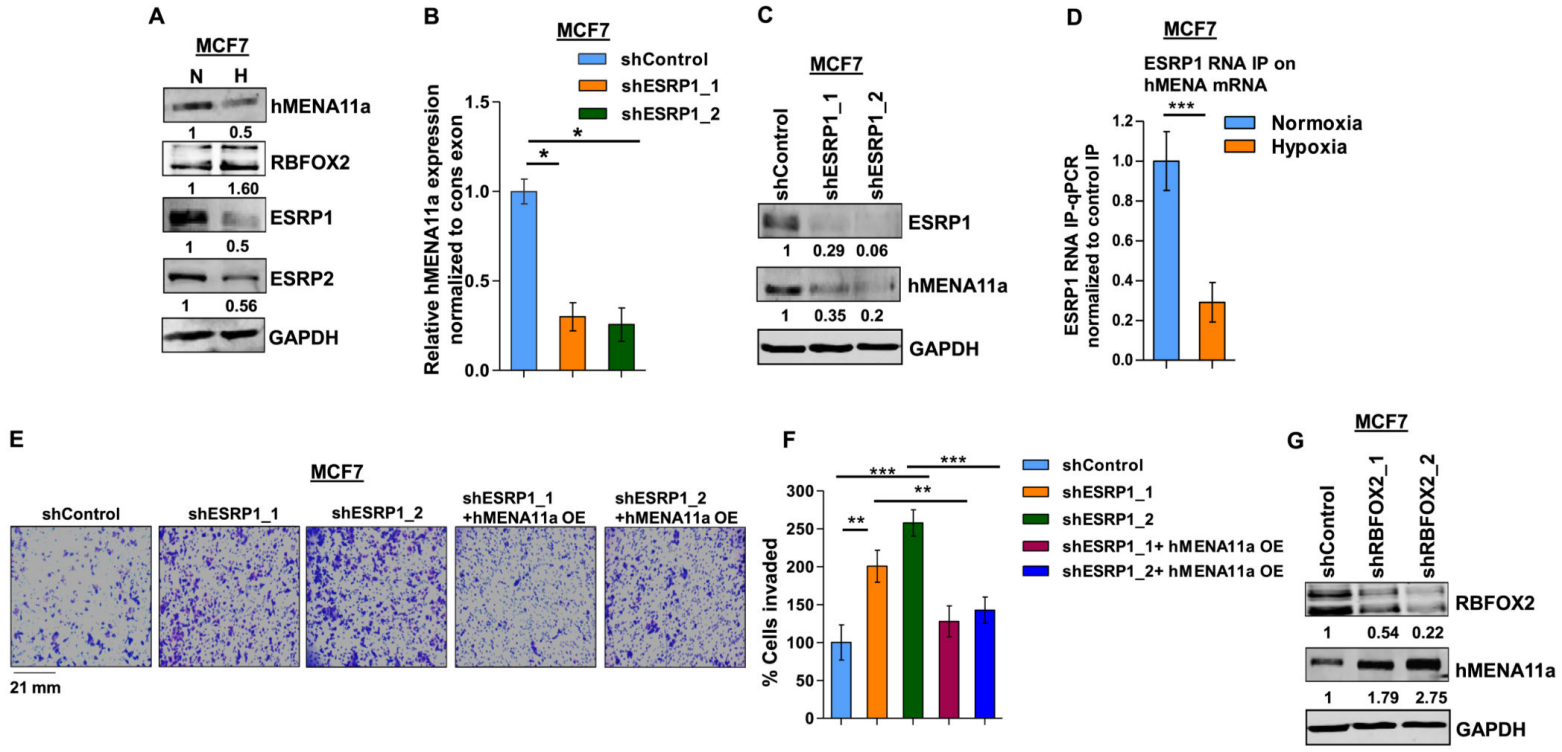
ESRP1 is downregulated under hypoxia and leads to exon 11a exclusion from *hMENA* pre-mRNA. (**A**) Immunoblot of hMENA11a, RBFOX2, ESRP1 and ESRP2 under hypoxia versus normoxia in MCF7. (**B**) qRT-PCR of *hMENA11a* isoform after ESRP1 knockdown in MCF7 under normoxic condition. (**C**) Immunoblot of ESRP1 and hMENA11a on ESRP1 knockdown in MCF7 under normoxic condition. (**D**) qRT-PCR of *hMENA* transcript after ESRP1 IP in MCF7 under normoxic and hypoxic conditions. Fold enrichment (ESRP1/IgG) was normalized to input. (**E**) Invasion assay and (**F**) its quantification in MCF7 and (**G**) immunoblot of RBFOX2 and hMENA11a after RBFOX2 knockdown in MCF7 under hypoxic condition. Error bars show mean values ± SD (*n* = 3 unless otherwise specified). As calculated using two-tailed Student’s *t*-test, *P < 0.05, **P < 0.01 and ***P < 0.001.

EMT is known to be induced under hypoxia via altered expression of a plethora of transcription and splicing factors (46). As far as splicing factors are concerned, the positive correlation of a low ESRP/RBFOX2 ratio with metastasis reported in various studies (6,47,48) led us to investigate their plausible role in hypoxia-induced *hMENA* splicing. Upon hypoxic stress, we observed a steep decline in ESRP expression and an increase in RBFOX2 expression in MCF7 (Figure 3A) and HCC1806 cells (Supplementary Figure S3A). To explore the involvement of the ESRPs in *hMENA* splicing, we performed an shRNA-mediated knockdown of *ESRP1* under normoxia and observed a drastic ablation of *hMENA11a* expression in both MCF7 (Figure 3B and C) and HCC1806 cells (Supplementary Figure S3B). This validates the role of ESRP1 in the inclusion of exon 11a, which is consistent with the decreased expression of *hMENA11a* under hypoxia. The inclusion of exon 11a was rescued when *ESRP1* was ectopically expressed under hypoxia (Supplementary Figure S3C–F). Besides, the downregulation of ESRP2 also inflicted the exclusion of exon 11a (Supplementary Figure S3G). Notwithstanding, ESRP1 appears to be more effective in protecting hMENA exon 11a, probably because ESRP2 is less robust than ESRP1 (49). Hence, further studies were performed only concerning ESRP1. To understand the binding of ESRP1 on *hMENA* transcript, we performed the RNA IP assay in MCF7 that indicated that the strong enrichment of ESRP1 decreased significantly under hypoxia (Figure 3D). We also observed that the invasiveness and actin stress fiber formation were increased when *ESRP1* was knocked down in MCF7 cells under normoxia, and this phenomenon was reversed when *hMENA11a* isoform was ectopically expressed (Figure 3E and F, and Supplementary Figure S3J). Similar results were also obtained in HCC1806 cells (Supplementary Figure S3H–J). Downregulation of ESRP1 and ectopic expression ofhMENA11a in these knockdown MCF7 and HCC1806 cell lines were confirmed by immunoblotting (Supplementary Figure S3K–M). These results confirm that the *ESRP1* downregulation under hypoxia is key to *hMENA* exon 11a exclusion. Previous reports have suggested that, unlike *ESRP1*, *RBFOX2* gets upregulated during the acquisition of EMT (6,48). Our data also show up-regulation of RBFOX2 under hypoxia in both MCF7 (Figure 3A) and HCC1806 (Supplementary Figure S3A). We went on to investigate the interplay between RBFOX2 and *hMENA* splicing by an shRNA-mediated knockdown of *RBFOX2* under hypoxia, which increased the expression of *hMENA11a* isoform in MCF7 cells (Figure 3G and Supplementary Figure S3N). This suggested that the presence of RBFOX2 enhances the cassette exon 11a exclusion under hypoxia. The detailed analysis of how RBFOX2 contributes to *hMENA* AS is discussed later (Figure 5).

### Hypoxia-induced upregulation of SLUG represses *ESRP1* expression

**Figure 4:**
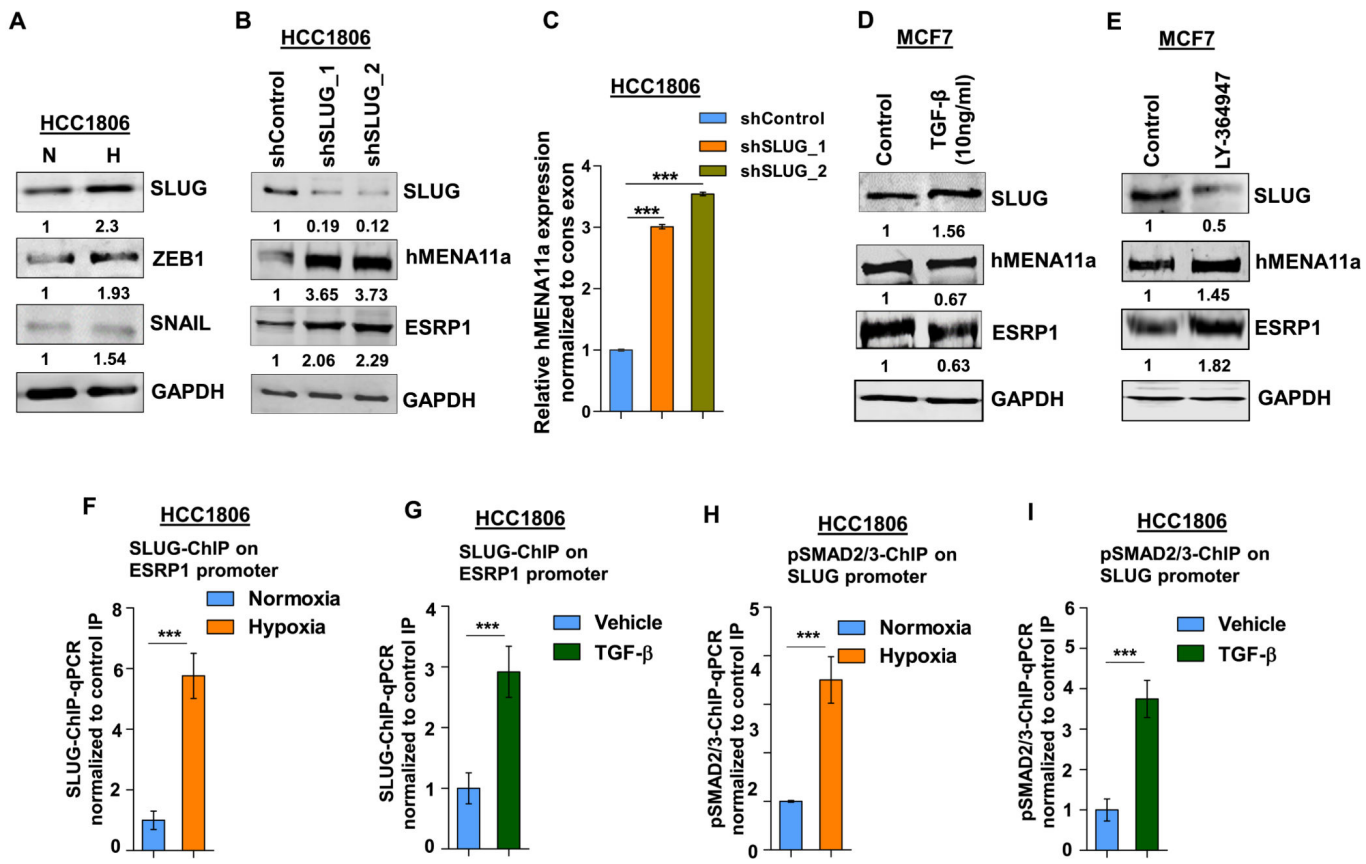
SLUG upregulation under hypoxia downregulates ESRP1 and causes exon 11a exclusion from *hMENA* pre-mRNA. (**A**) Immunoblot of SLUG, ZEB1 and SNAIL under hypoxia versus normoxia in HCC1806. (**B**) Immunoblot of SLUG, hMENA11a and ESRP1 and (**C**) qRT-PCR of *hMENA11a* isoform after SLUG knockdown in HCC1806 under hypoxic condition. Immunoblot of SLUG, hMENA11a and ESRP1 after (**D**) TGF-β (10 ng/ml) (under normoxia) and (**E**) TGF-β inhibitor treatment (under hypoxia) in MCF7. ChIP qRT-PCR on ESRP1 promoter using SLUG antibody in normoxia and hypoxia (**F**), and TGF-β treatment (**G**) in HCC1806 cells. Fold enrichment (SLUG/IgG) was normalized to 5% input. ChIP qRT-PCR on SLUG promoter using pSMAD2/3 antibody in normoxia and hypoxia (**H**), and TGF-β treatment (**I**) in HCC1806 cells. Fold enrichment (pSMAD2/3/IgG) was normalized to 5% input. Error bars show mean values ± SD (*n* = 3 unless otherwise specified). As calculated using two-tailed Student’s *t-test,* **P < 0.01 and ***P < 0.001.

Next, we wanted to characterize the transcriptional repressors that downregulate the expression of *ESRP1* and eventually modulate *hMENA* splicing. Previous studies have reported the role of transcription repressors SLUG, SNAIL and ZEB1 in the acquisition of EMT (50). We observe that the SLUG and ZEB1 were consistently upregulated in HCC1806 (Figure 4A) and MCF7 (Supplementary Figure S4A) cells under 24 h of hypoxia treatment. Further, to verify whether SLUG and ZEB1 transcriptionally modulate the expression of splicing factors and thereby modulate *hMENA* splicing, knockdown experiments were performed. We observed that the shRNA-mediated *SLUG* knockdown, but not ZEB1 knockdown (Figure 4B and C, and Supplementary Figure S4B–D), showed an increase in *ESRP1* and *hMENA11a* expression. To examine whether hypoxia-driven TGF-β signaling activation leads to *SLUG* upregulation, we treated HCC1806 cells with TGF-β ligand (10 ng/ml) under normoxia. It was observed that TGF-β treatment led to an upregulation of SLUG, and downregulation of ESRP1 and hMENA11a (Figure 4D and Supplementary Figure S4E). These phenomena were reversed when the cells were treated with TGF-β inhibitor under hypoxia (Figure 4E and Supplementary Figure S4F). These results indicate that upon getting upregulated via hypoxia-induced TGF-β signaling, SLUG might act as a transcriptional repressor of *ESRP1.* Next, we confirmed the binding of SLUG on *ESRP1* promoter under hypoxia and upon TGF-β treatment by ChIP. Both the treatments showed significant enrichment of SLUG on the *ESRP1* promoter as compared to the controls (Figure 4F and G). To further prove that hypoxia-mediated upregulation of *SLUG* is orchestrated by TGF-β signaling, we checked for the enrichment of pSMAD2/3 on *SLUG* promoter by ChIP upon hypoxia as well as upon TGF-β treatment. Both the treatments showed a significantly enhanced enrichment of pSMAD2/3 on the *SLUG* promoter (Figure 4H and I). This suggests that the hypoxia-induced TGF-β pathway upregulates the expression of *SLUG.*

Furthermore, we also observed that the knockdown of *SLUG* under hypoxic conditions leads to the reduced invasion of breast cancer cells, which was reversed by both over-expression of *hMENAΔ11a* isoform and *ESRP1* knockdown in the *SLUG-dep*le*ted* cells (Supplementary Figure S4G-K). Collectively, these results indicate that *SLUG*is induced by hypoxia-driven TGF-β signaling, and it transcriptionally downregulates *ESRP1,* which eventually results in an abatement of hMENA11a isoform.

### Hypoxia-driven TGF-β signaling upregulates RBFOX2 that in turn transcriptionally represses *ESRP1*

**Figure 5:**
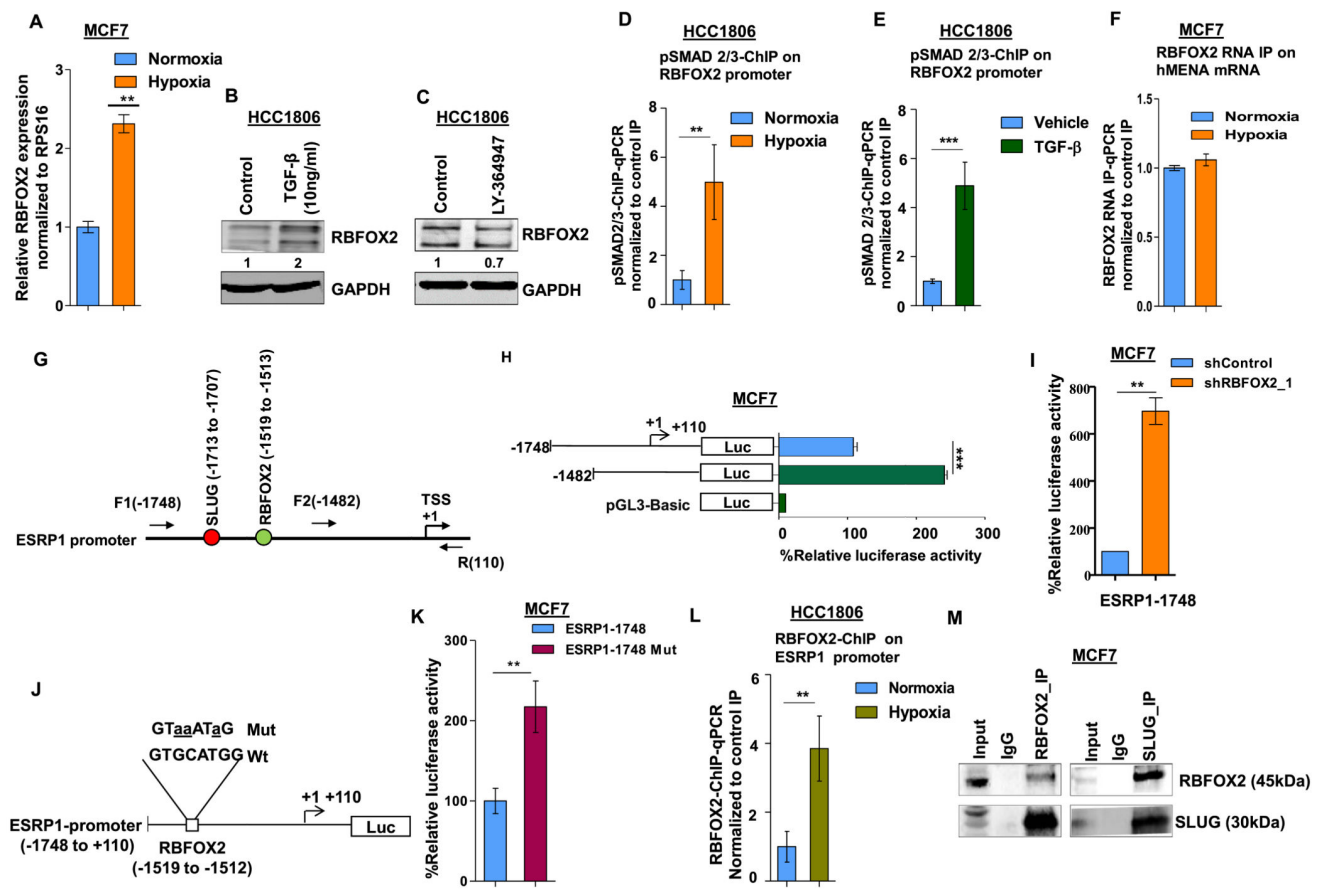
Hypoxia-driven TGF-β signaling upregulates RBFOX2, which in turn negatively regulates ESRP1 expression. (**A**) qRT-PCR for *RBFOX2* under normoxia and hypoxia in HCC1806 (Ct values are normalized to RPS16). Immunoblot for RBFOX2 expression in HCC1806 cells after (**B**) TGF-β and (**C**) inhibitor (LY-364947) treatment under normoxia and hypoxia, respectively (GAPDH was used as a control). ChIP qRT-PCR on RBFOX2 promoter using pSMAD2/3 antibody in (**D**) normoxia and hypoxia, and (**E**) TGF-β treatment in HCC1806 cells. Fold enrichment (pSMAD2/3/IgG) was normalized to 5% input. (**F**) RNA IP for RBFOX2 binding on *hMENA* transcript in normoxic and hypoxic conditions in MCF7. Fold enrichment (Rbfox2/IgG) was normalized to input. (**G**) Schematic representation of the *ESRP1* promoter and the positions of primers are indicated. SLUG and RBFOX2 binding sites on *ESRP1* promoter are indicated in red and green colors, respectively; numbers indicate the positions of SLUG and RBFOX2 binding sites. +1 over the arrow indicates TSS. (**H**) Deletion constructs of the *ESRP1* promoter and their luciferase activities in MCF7 cells under hypoxia. Various lengths of *ESRP1* promoters were generated by PCR and inserted into reporter vector pGL3. (**I**) After the transfection of *RBFOX2* shRNA or shControl, MCF7 cells were incubated under hypoxia for 24 h. Thereafter, *ESRP1-1748* reporter plasmids were transfected and incubated for 24 h, and the reporter assays were carried out. The relative luciferase values are shown as mean ± SD. (**J**) Schematic representation of the *ESRP1*-1748/luciferase reporter constructs. The *RBFOX2* (−1748/−110 bp) sequences for both wild-type and mutated constructs are shown; mutated nucleotides are represented by lowercase letters and underlined. (**K**) Wild-type or mutant *RBFOX2* luciferase reporter constructs were co-transfected with the Renilla luciferase vector in MCF7 cells under hypoxia, and the luciferase activity was measured after 24 h of transfection. The relative luciferase values are shown as mean ± SD. (**L**) ChIP qRT-PCR on *ESRP1* promoter using the RBFOX2 antibody in normoxia and hypoxia in HCC1806 cells. Fold enrichment (RBFOX2/IgG) was normalized to 5% input. (**M**) The endogenous RBFOX2 and SLUG proteins interact in MCF7 cells. IP was performed with RBFOX2 or SLUG antibodies under hypoxia, and IgG was used as a control. The immunoprecipitated samples were analyzed by immunoblotting using the SLUG antibody and RBFOX2 antibody. Error bars show mean values ± SD (*n* = 3 unless otherwise specified). As calculated using two-tailed Student’s *t*-test, *P < 0.05, **P < 0.01 and ***P < 0.001.

In the previous section, we have shown that *RBFOX2* gets upregulated upon hypoxia treatment (Figure 3A and Supplementary Figure S3A). The same phenomenon has been validated by qRT-PCR in MCF7 cells (Figure 5A). To investigate whether hypoxia-induced *RBFOX2* expression (Figure 5A) is mediated by TGF-β signaling, normoxic cells were treated with TGF-β ligand that resulted in RBFOX2 upregulation (Figure 5B). Likewise, TGF-β inhibitor treatment under hypoxia, as expected, reduced RBFOX2 expression (Figure 5C). To assess the direct role of TGF-β signaling in RBFOX2 expression, we further performed the ChIP assay using the pSMAD2/3 antibody under hypoxia as well as upon treatment with TGF-β. Both the treatments exhibited enhanced enrichment of pSMAD2/3 on the *RB-FOX2* promoter as compared to control (Figure 5D and E), confirming the role of TGF-β signaling in the upregulation of *RBFOX2* under hypoxia. To identify how RBFOX2 affects *hMENA* splicing, we performed the RNA IP assay and observed that the difference in the occupancy of RBFOX2 on *hMENA* transcript under hypoxia versus normoxia was insignificant (Figure 5F). This finding insinuated that RB-FOX2 might promote *hMENA* exon 11a splicing indirectly by regulating other splicing factors, for instance, ESRP1. We went on to investigate the possible involvement of RB-FOX2 in the splicing switch of *ESRP1* transcript under hypoxia from a set of isoforms containing NLS (NLS+) to the isoforms devoid of NLS (NLS–) (51). Semi-quantitative and qRT-PCR experiments with appropriate primers (Supplementary Table S7) showed no significant turnaround in the isoform type as the expression of both NLS+ and NLS–isoforms of *ESRP1* was repressed under hypoxia (Supplementary Figure S5A and B). The shRNA-mediated knockdown of RBFOX2 under hypoxia increased the expression of both isoforms (Supplementary Figure S5C and D), suggesting an inhibitory role of RBFOX2 at the transcription level rather than at the splicing level of *ESRP1*. In a few recent studies, proteins involved in RNA processing have been appreciated for their entanglement in various stages of gene expression aside from their canonical roles in RNA splicing (52–54). In 2017, Wei *et al*. showed that RBFOX2 can regulate gene expression in a nascent RNA-dependent manner in conjunction with polycomb complex 2 (55). To investigate the role of RBFOX2 as a transcriptional repressor of *ESRP1*, we transfected MCF7 cells with the luciferase reporter construct of full-length *ESRP1* promoter (−1748), as well as of a truncated *ESRP1* promoter that is devoid of a putative RBFOX2 binding site (Figure 5G). The luciferase activity is highly elevated in cells transfected with luciferase construct of truncated promoter as compared to those with full-length promoter under hypoxic condition (Figure 5H), which is indicative of the transcriptional repressor activity of RBFOX2. In line with this observation, the shRNA-mediated knockdown of *RBFOX2* showed increased luciferase activity as compared to control shRNA (Figure 5I) in cells transfected with the full-length *ESRP1* promoter–luciferase construct. To further validate the transcriptional repressor activity of RBFOX2, we further created luciferase reporter constructs of *ESRP1* promoter wherein the RB-FOX2 binding site (TGCATG) is disrupted by site-directed mutagenesis (Figure 5J) (56,57). Upon transfection, the mutated construct showed higher luciferase activity as compared to its wild-type counterpart (Figure 5K). Moreover, the ChIP assay performed with the anti-RBFOX2 antibody revealed a remarkable enrichment of RBFOX2 at *ESRP1* promoter under hypoxia (Figure 5L). Similar to RBFOX2, the knockdown of *SLUG* enhanced the luciferase activity in cells housing the full-length *ESRP1* promoter–luciferase construct (Supplementary Figure S5E). Furthermore, the co-IP assay revealed a strong interaction of RBFOX2 with SLUG (Figure 5M). We also investigated the possible interdependence of SLUG and RBFOX2 to repress *ESRP1* expression. Engagingly, the shRNA-mediated knockdown of either of SLUG and RBFOX2 decreased the occupancy of its partner on ESRP1 promoter (Supplementary Figure S5F–I). These studies en masse attribute RBFOX2, for the first time, to a novel pursuit, as a transcriptional repressor of *ESRP1*. Additionally, to investigate the genome-wide binding of RBFOX2 to the gene promoters, we obtained the closest RBFOX2 ChIP-seq peak (obtained from the dataset GSE106042) corresponding to the TSSs of all human genes and calculated the genomic distance between them. We found that several genes belonging to various pathways like mRNA splicing, translation and DNA repair, among others, were having RBFOX2 ChIP-seq peaks within 2 kb of their TSSs (Supplementary Table S11 and Supplementary Figure S5J). The occupancy profile of RB-FOX2 around a region of 2 kb of TSS and the distribution of the peaks on several regions in genome showed that a remarkable number of peaks were coinciding with the gene promoters (Supplementary Figure S5K and L). This indicates that binding of RBFOX2 to genomic DNA could be widespread and that it might play a role in governing the expression of several genes.

### Clinical validation of hypoxia-induced TGF-β–ESRP1–hMENA11a axis

**Figure 6:**
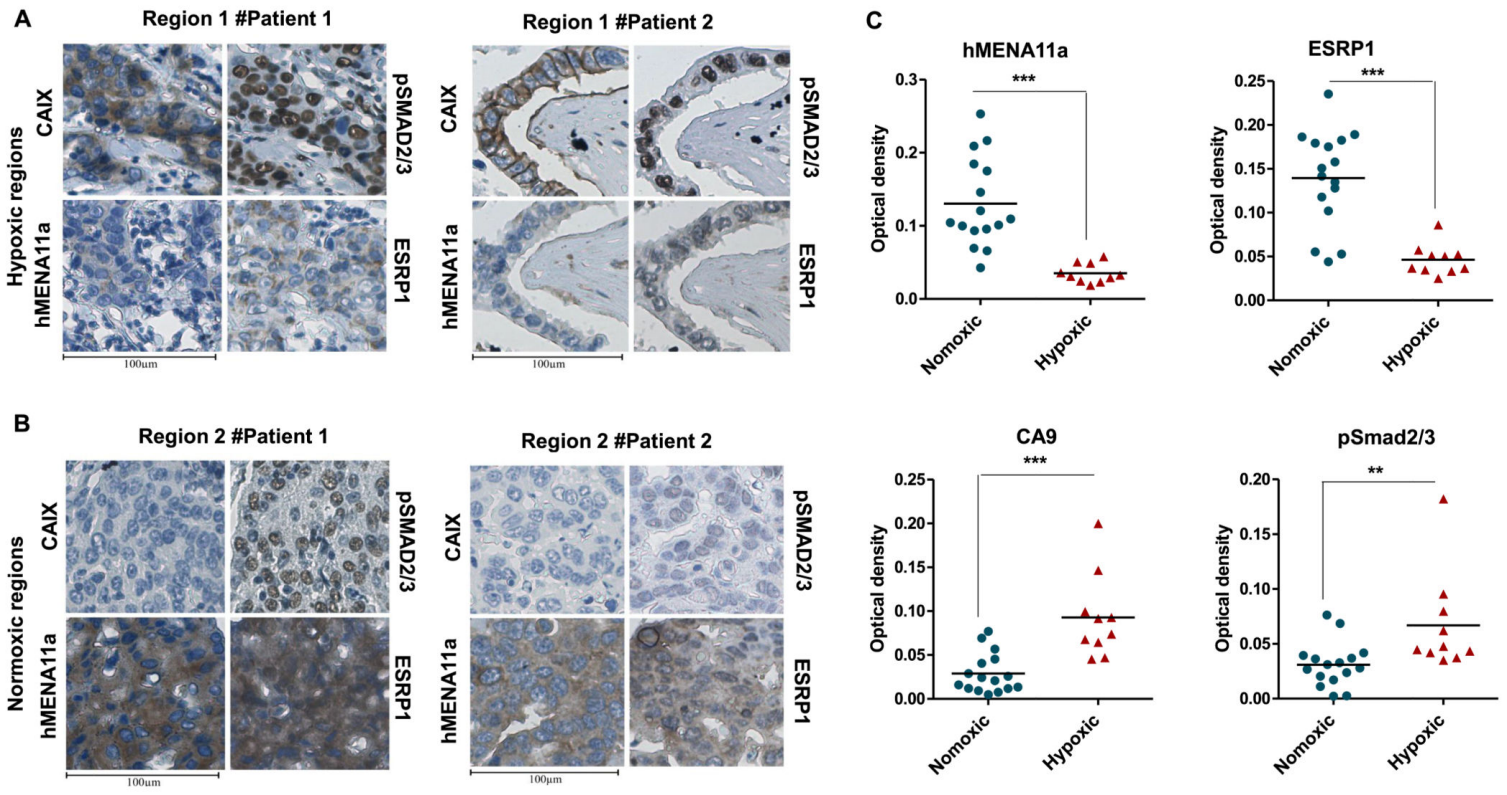
(**A, B**) CAIX, pSMAD2/3, hMENA11a and ESRP1 immunostaining of two illustrative cases of breast cancer patients. (**A**) Hypoxic regions of patients 1 and 2. Hypoxic regions: areas representing strong membranous and/or cytoplasmic immunostaining for CAIX also exhibit strong expression of pSMAD2/3 (nuclear) and weak expression of hMENA11a and ESRP1 (cytoplasmic). (**B**) Normoxic regions of patients 1 and 2. Normoxic regions: areas representing weak immunostaining for CAIX also exhibit weak expression of pSMAD2/3 and strong expression of both hMENA11a and ESRP1. Magnification: 40×. (**C**) Quantification for hMENA11a, ESRP1, CAIX and pSmad staining represented as optical density for hypoxic versus normoxic regions of 19 breast cancer patients.

To investigate the association between hypoxia and hMENA11a *in vivo,* we performed immunohistological analyses of breast tissue sections obtained from breast cancer patients. Since carbonic anhydrase IX (CAIX) has been previously identified as a biomarker of hypoxia (58), the sections were immunostained with CAIX antibody in parallel with hMENA11a, ESRP1 and pSMAD2/3. The regions corresponding to positive CAIX staining showed weak ESRP1 and hMENA11a expression and strong pSMAD2/3 expression (Figure 6A, and Supplementary Figure S6A, C and G). Notably, normoxic regions (negatively stained for CAIX) of the tumor tissue correspond to strong hMENA11a and ESRP1 expression and reduced pSMAD2/3 expression (Figure 6B, and Supplementary Figure S6B, D, E and F). Hematoxylin and eosin staining for patient samples is shown in Supplementary Figure S6H. The DAB staining when quantified for all 19 patients shows increased staining for CAIX and pSMAD in hypoxic compared to normoxic regions (even in the same patient), while it shows decreased staining for ESRP1 and hMENA11a (Figure 6C). These results corroborate with our findings in the breast cancer cell lines that hypoxic tumor cells are associated with increased pSMAD2/3 and decreased ESRP1 and hMENA11a expression, while normoxic cells are associated with strong ESRP1 and hMENA11a expression. Taken together, these clinicopathological observations support the role of hypoxia-induced TGF-β signaling in mediating the hMENA AS and thereby cancer progression.

### Hypoxia-induced TGF-β signaling modulates global AS of EMT-associated genes

**Figure 7:**
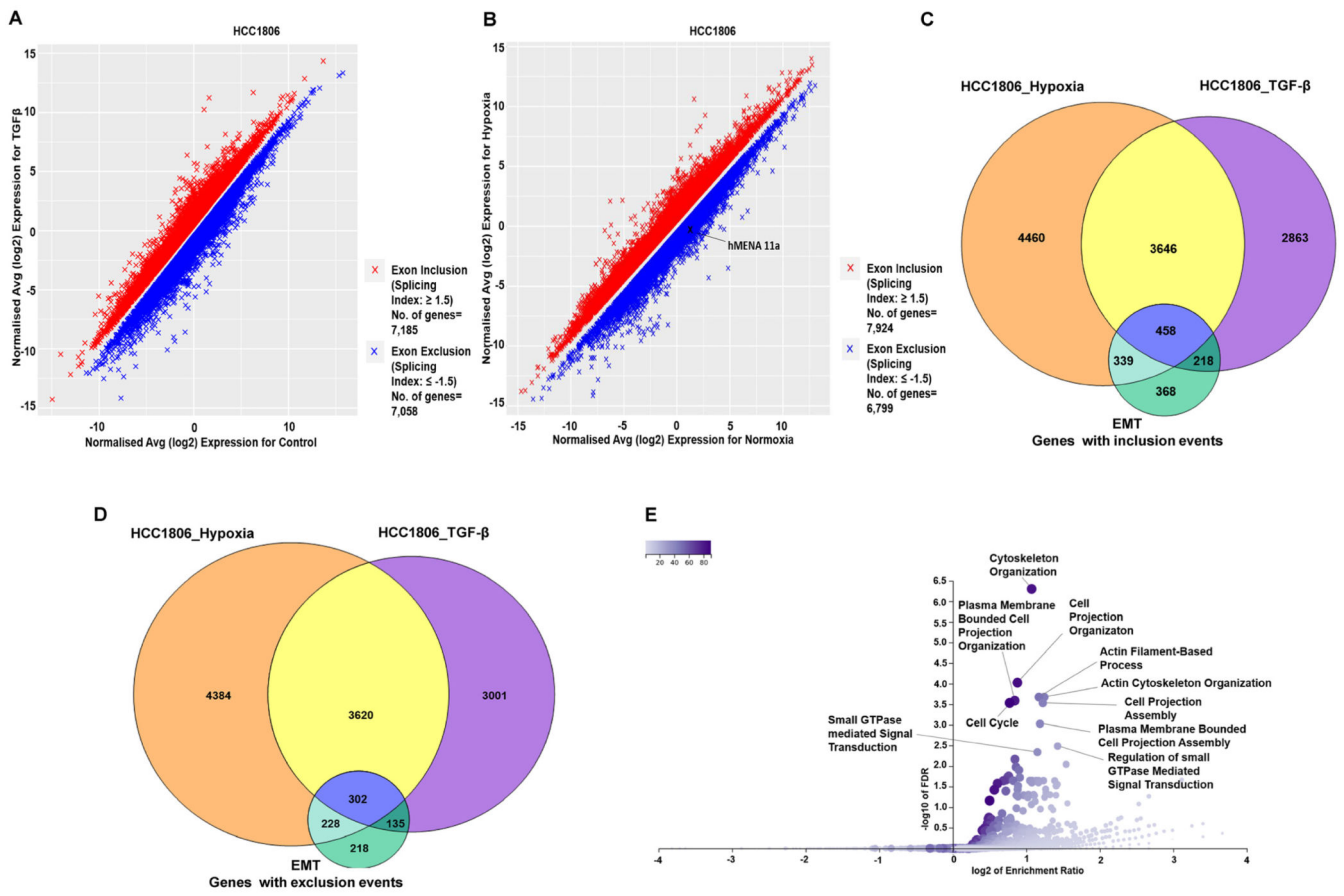
The global effect of TGF-β signaling on AS under hypoxia in breast cancer cell line HCC1806. (**A**) Microarray HTA 2.0 profile showing differential splicing events in TGF-β-treated versus control HCC1806 cells (*n* = 2, |SI| ≥ 1.5, *P <* 0.05). (**B**) Microarray HTA 2.0 profile showing the differential splicing events in hypoxia-treated (cells cultured in 1% O_2_) versus control (normoxic) HCC1806 cells (*n* = 2, |SI| ≥ 1.5, *P <* 0.05). (**C**) The common genes that show significant exon inclusion events on TGF-β treatment, hypoxia induction and during EMT in HCC1806. A total of 4104 genes show exon inclusion in both hypoxia and TGF-β treatment, while 458 genes show inclusion events in all the three conditions. (**D**) Common genes that show significant exon exclusion events on TGF-β treatment, hypoxia induction and during EMT in HCC1806. A total of 3922 genes show exclusion events under both hypoxia and TGF-β induction, while 302 genes show exon exclusion under all three conditions. (**E**) Volcano plot representing the enrichment ratio of various GO terms over-represented in the set of genes (*n* = 760) that show either exon inclusion or exclusion in all three conditions (FDR < 0.05).

After establishing the crucial role of TGF-β signaling in mediating the splicing switch of *hMENA* under hypoxia, we went on to investigate TGF-β-induced global changes in the AS program of breast cancer cells. In MCF7 and HCC1806 cell lines, we performed microarray profiling experiments using the HTA 2.0 array to look for the splicing changes in cells treated with TGF-β ligand compared to control cells. Additionally, HTA 2.0 profiling was also performed with hypoxia-treated cells compared to normoxic cells. The splicing events with an |SI| value of ≥1.5 and *P <* 0.05 were considered to be statistically significant and are represented in Figure 7A and B, and Supplementary Figure S7A–D. The differential splicing of cassette exons upon hypoxia exposure, corresponding to a few representative genes (KDM5B, USP47, DLG1 and PLOD2), was confirmed by qRT-PCR (Supplementary Figure S7E and F). Confining the analysis to events that are relevant to our hypothesis, we looked for the overlap among splicing changes after TGF-β induction, hypoxia treatment and those associated with EMT (20). In HCC1806, we found that out of a total of 7185 genes that showed significant exon inclusion events after TGF-β treatment, 4104 genes get spliced in the same direction under hypoxia as well. Moreover, 458 genes that showed exon inclusion during EMT also overlap with the aforesaid conditions (Figure 7C). Furthermore, out of the 7058 genes that undergo exon exclusion events after TGF-β induction in HCC1806, 3922 genes showed the same pattern when treated with hypoxia. The 302 genes that showed exon exclusion during EMT also intersect with both hypoxia- and TGF-β-treated conditions (Figure 7D). In line with our observation with HCC1806, MCF7 cells also show a comparable overlap among hypoxia-induced, TGF-β-induced and EMT-associated splicing profiles (Supplementary Figure S7G and H, and Supplementary Table S3). These findings suggest that the change in AS events influenced by TGF-β signaling under hypoxia is not just confined to hMENA but instead is a large-scale phenomenon. Furthermore, ORA was performed with the set of common genes that showed either exon inclusion or exclusion in all three conditions. We found that GO terms for biological processes such as cell projection organization, cytoskeleton organization and cell projection assembly, among others, were enriched in gene sets from both MCF and HCC1806 in theORA (Figure 7E and Supplementary Figure S7I). These GO terms are indicative of the transition of breast cancer cells to a more migratory and invasive phenotype upon the induction of TGF-β signaling, further reinforcing its association with the global changes in AS of EMT-associated genes under hypoxia.

## Discussion

There is a growing appreciation that hypoxic milieu present in the core of solid tumors plays a crucial role in cancer progression by effectuating EMT (38). Previous studies have identified a substantive change in the AS profile of EMT-associated genes (6). However, whether and how hypoxia contributes to AS events leading to EMT lacks clarity. It is noteworthy that AS of exon 11a of hMENA, which regulates the actin remodeling during EMT, is vastly deregulated in many cancer types (15,16). The splicing switch from hMENA11a to hMENAΔ11a in cancer cells supports rapid actin polymerization that in turn promotes invasiveness and migration (17). In this study, for the first time, we divulge the molecular insight about how low-oxygen stress brings about AS of*hMENA* in breast cancer. A myriad of signaling pathways are known to be deregulated in tumor hypoxia (46). We report that activation of canonical TGF-β signaling under hypoxia plays a substantial role in the AS of *hMENA.*

Moreover, studies have shown that the TGF-β signaling is stimulated in cancer cells by TGF-β cargo packaged in exosomes (59). Besides, cancer cells are reported to release a greater amount of exosomes under hypoxic stress in an HIF-dependent manner (45). Our present study also corroborated the elevated exosome release by breast cancer cells under low-oxygen tension. Further, we report that the hypoxic exosomes contain greater quanta of TGF-β as against normoxic exosomes. Subsequently, the treatment with hypoxic exosomes resulted in *hMENA* exon 11a exclusion by triggering TGF-β signaling. We believe that the HIF-dependent nature of exosome production under hypoxia could be one major reason behind the HIF dependence of hMENA AS. Remarkably, this is the very first instance where the potential of hypoxic exosomes to drive the AS has been established. This finding lays the foundation for future studies on the prospective involvement of exosomes to rewire cancer-associated AS events.

EMT has widely been correlated with the upregulation of TFs such as SLUG, SNAIL, ZEB1 and TWIST (50). We report that hypoxia-elicited TGF-β signaling enhances the expression of SLUG that subsequently constrains the expression of ESRP1, which governs *hMENA* exon 11a splicing. This happens to be the first study describing the SLUG-mediated transcriptional regulation of *ESRP1.* Although, besides ESRP1 splicing factor, RBFOX2 has been associated with *hMENA* AS regulation (47), the precise molecular mechanism was not reported. Intriguingly, we observed that RBFOX2 does not regulate *hMENA* AS by directly binding to its pre-mRNA. Rather, it acts as a transcriptional repressor of *ESRP1* while strongly interacting with SLUG under hypoxia. Genome-wide analysis of ChIP-seq data (obtained from the dataset GSE106042) revealed that a remarkable number of genes had RBFOX2 ChIP-seq peaks within 2 kb of their TSSs. These findings envision that RBFOX2 can potentially be involved in the transcriptional regulation of a range of other genes synergistically with TFs and epigenetic modifiers.

**Figure 8:**
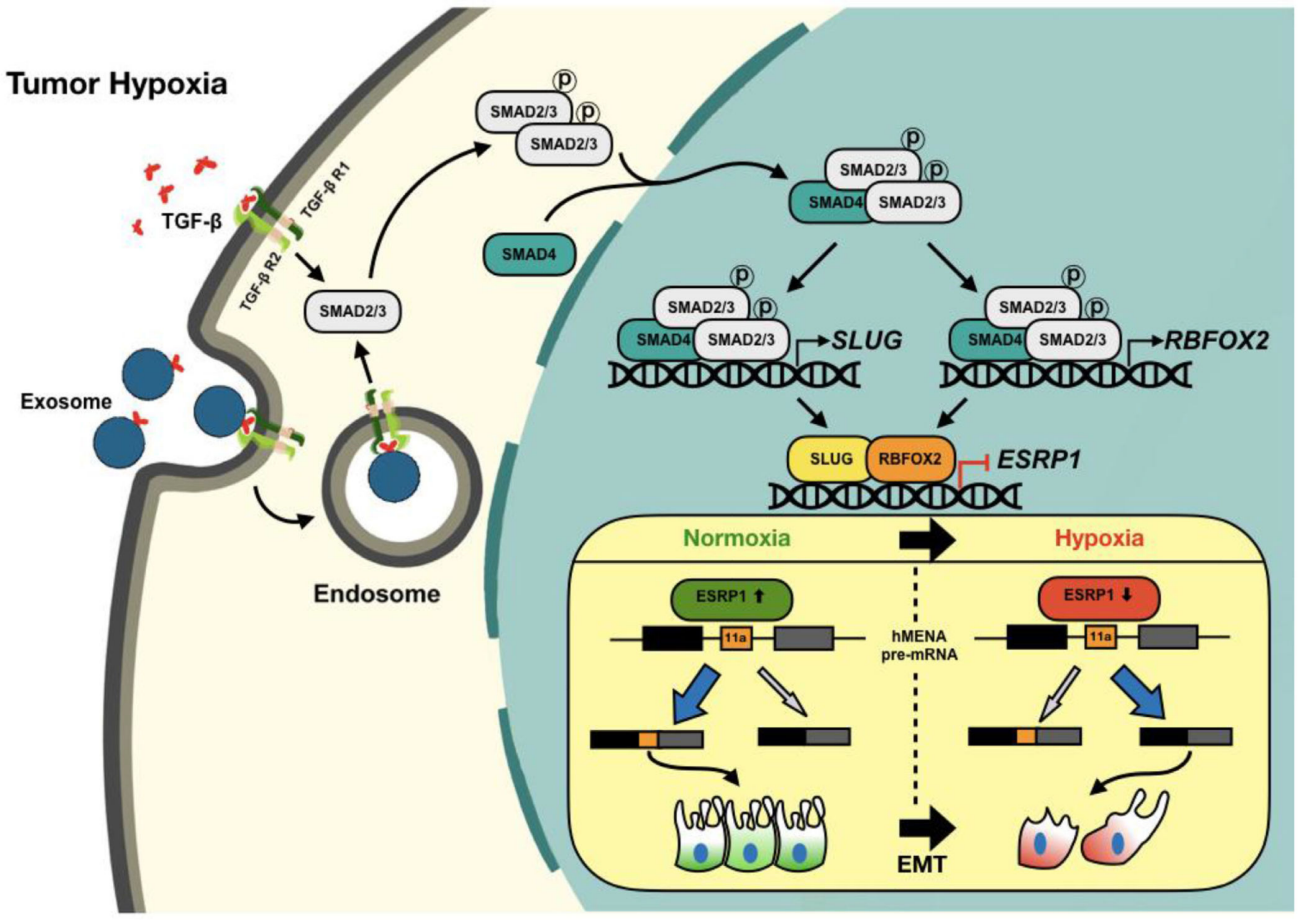
Schematic representation of hypoxia-driven TGF-β signaling to mediate AS of hMENA exon 11a. The TGF-β signaling is stimulated under hypoxia, effectuating the phosphorylation of SMAD2 and/or SMAD3 proteins. Hypoxic exosomes contribute to the actuation of TGF-β signaling. Phos-phorylated SMAD2/3 form a complex with their constitutive partner SMAD4. The activated SMAD protein complex translocates to the nucleus and enhances SLUG and RBFOX2 expression. SLUG and RBFOX2 transcriptionally repress ESRP1 while interacting with each other. Under the normoxic condition, ESRP1 promotes the inclusion of exon 11a of hMENA in the final transcript that supports epithelial phenotype. A reduced level of ESRP1 under hypoxia leads to the production of hMENAΔ11a isoform that gives rise to the mesenchymal phenotype under hypoxia and thereby promotes cell invasion.

Moreover, our clinicopathological results substantiate the role of hypoxia-driven TGF-β signaling for *hMENA* AS in breast cancer tissues. The global analysis of splicing changes during hypoxia-induced TGF-β signaling activation reveals a large-scale rewiring of AS programs in EMT-related genes. Collectively, this body of work provides the mechanistic foundation for how hypoxia drives *hMENA* AS in breast cancer (Figure 8), and suggests the relevance of hypoxia-driven TGF-β signaling and its multifaceted aspects in the potential development of novel anti-EMT therapeutic approaches.

## Supplementary Material

Supplementary Data are available at NAR Cancer Online.

Supplementary Table 1

Supplementary Table 2

Supplementary Table 3

Supplementary Table 4

Supplementary Table 5

Supplementary Table 11

Supplementary data

## Data Availability

The results of the microarray analyses were deposited in GEO under accession number GSE147516.
