# Supplementary material for: Hypoxia-induced TGF-β–RBFOX2–ESRP1 axis regulates human MENA alternative splicing and promotes EMT in breast cancer
Source: NAR Cancer. 2020 Sep 18;2(3):zcaa021. doi: 10.1093/narcan/zcaa021 (PMC7116222; doi:10.1093/narcan/zcaa021)
Supplement: zcaa021_Supplemental_Files [file zcaa021_supplemental_files.zip › NAR Cancer Supplementary data.pdf]

Hypoxia-induced TGF-β-RBFOX2-ESRP1 axis regulates human MENA alternative splicing and promotes EMT in breast cancer

Neha Ahuja[1,†], Cheemala Ashok [1,†], Subhashis Natua [1], Deepak Pant [1], Anna Cherian[1], Madhura R Pandkar [1], Pooja Yadav [1], Vishnu Narayanan S.S. [1], Jharna Mishra[2], Atul Samaiya[3], Sanjeev Shukla[1,*]

[1]Department of Biological Sciences, Indian Institute of Science Education and Research, Bhopal, Madhya Pradesh 462066, India; [2]Department of Oncopathology, Bansal Hospital (BH), Bhopal, Madhya Pradesh 462016, India; [3]Department of Surgical Oncology, BH, Bhopal, Madhya Pradesh 462016, India

*To whom correspondence should be addressed. Email: sanjeevs@iiserb.ac.in

[†]These authors contributed equally.

<u>Supplementary Table 6:</u> List of antibodies utilized for Immunohistochemistry, RNA Immunoprecipitation, and Immunoblotting.

**S.No**	**Antibody**	**Company**	***Catalog* no**	**Lot .no**
1	pan-hMENA	Sigma	HPA028696	A104529
2	GAPDH (D16h11)	CST	5174S	7
3	Human phospho-smad2/3 (S465/S467)	R&D Systems	MAB8935	CJRA0318051
4	Anti slug	Sigma	PRS3959	87711801
5	hMENA11a	Sigma	HPA028448	A104733
6	RBM35A(ESRP1)	Abcam	ab107278	GR279719-9
7	Fox2 / RBM9	Abcam	ab57154	GR317972-9
8	ESRP2	Abcam	ab113486	GR322407-11
9	[EPR5702] to CD63	Abcam	ab134045	GR3212162-1
10	TCF8/ZEB1 (D80D3)	CST	3396S	9
11	Snail (C15D3)	CST	3879S	12
12	HIF-1α (D2U3T)	CST	14179S	3
13	TGF-BETA1,-2,-3	R&D Systems	mab1835-100	CCL1218082
14	Anit-Flag tag	Novus Biologicals	NBP1-06712SS	B-6
15	Anti E cadherin	Abcam	Ab40772	GR148899-1
16	Anti N Cadherin	Abcam	ab19348	GR306928-1
17	Anti Vimentin	Abcam	ab137321	GR294886-5
18	Alexa-Flour 680 anti-rabbit IgG	Invitrogen	A32734	RJ243414
19	Alexa-Flour 800 anti-mouse IgG	Invitrogen	A32730	SC243837
20	Alexa-Flour 488 anti-mouse IgG	Invitrogen	A11059	1832425

21	Alexa-Flour 555 anti-rabbit IgG	Invitrogen	A32732	1858260
22	Anti Carbonic Anhydrase	Abcam	ab184006	GR173128-25
23	Alexa-Flour 680 anti-rat IgG	Invitrogen	A21096	2010149

Supplementary Table 7: List of primer sequences utilized for qPCR and RNA Immunoprecipitation

S.No	Primers	Sequence
1	RBFOX2 Fw	CTCACCCAGCACACAAAATG
2	RBFOX2 Rev	ACTGCTGGCCGTCTGTCT
3	hMENA ex11a Fw	ACG GGA TTC TCC AAG GAA AAA TCA
4	hMENA ex13 Rev	CTGCTTCAGCCTGTCATAGTCAA
5	hMENA ex3 Fw	AATCAAGCTACACAGACCTTCC
6	hMENA ex3 Rev	ATGGCACTTGCGAAGACA
7	hMENA ex5/6 Fw(junc)	ACAGGAAACAGCCCAGAGCAAG
8	hMENA ex6 Rv	GAACTTGAGCAGGTAGTTGTGAGTTTTGT
9	ESRP1 ex2 Fw	GCTAGTTAGACCGGATCAGTT
10	ESRP1 ex2 Rev	CCGAGGACAGGCTTTCG
11	ESRP1ex12 Fw	GCCATGTAAGTTACCATGCC
12	ESRP1ex12/13 Fw	CCCACCGCCATGCCTGTCT
13	ESRP1 ex14 Rev	CCAAGACTATTAGGCGAACC
14	RPS16 Fw	AAA CGC GGC AAT GGT CTC ATC AAG
15	RPS16 Rev	TGG AGA TGG ACT GAC GGA TAG CAT

16	hMENA11a ex9 Fw(SemiQ)	GAATTGCTGAAAAGGGATC
17	hMENA11a ex15 Rv(SemiQ)	CTGTTCCTCTATGCAGTATTTGAC
18	KDM5B ex6-Fw	AGACAGAGTCTCGCTGTGTTG
19	KDM5B ex7-Rv	TCTGGCTTCCGTTGTCTCCT
20	KDM5B ex7-Fw	AGACGTCGAATGGGTTGTCC
21	KDM5B ex9-Rv	CAGACATACAGGTCCACAGC
22	USP47 ex2-Fw	GATGTGTTTTGGAGATGCAGAC
23	USP47 ex3-Rv	AGACTCTAGGTTCTTCAGCA
24	USP47 ex4-Fw	GCACCACTGGATCATACCAG
25	USP47 ex4/5-Rv	AGCACTGGAATCCTCCAGCA
26	DLG1 ex5-Fw	CAGAGATTGAGAATGTCCATGG
27	DLG1 ex6/7-Rv	GAGGATTTGCCTGTGGTATG
28	DLG1 ex3-Fw	CAGAGCAACCTCTTTCAGGC
29	DLG1 ex4-Rv	CTGCTTGGCAGTGTCTCTGA
30	PLOD2 ex13-Fw	GGAAAGACACTCCGATCAGAG
31	PLOD2 ex14/15-Rv	ATACACCCTTTGGGGGGCTG
32	PLOD2 ex11-Fw	GCCGTCAGGATGAAAAGTGTG
33	PLOD2 ex12-Rv	CCATCAGGACTCAATGCTCC

<u>Supplementary Table 8:</u> Oligo sequence of shRNAs

shSLUG_1	5'-CCGGGCGCCCTGAAGATGCATATTCCTCGAGGAATATGCATCTTCAGGGCGCTTTTTG-3'
shSLUG_2	5'-CCGGCCGAAGCCAAATGACAAATAACTCGAGTTATTTGTCATTTGGCTTCGGTTTTTG-3'
shESRP1_1	5'-CCGGCTTGCAGCAAGATGGAACTTACTCGAGTAAGTTCCATCTTGCTGCAAGTTTTTG-3'
shESRP1_2	5'-CCGGGCATAAAGACTTGTTGGGTAACTCGAGTTACCCAACAAGTCTTTATGCTTTTTTG-3'
shESRP2_1	5'-CCGGGCCTTAAAGATCCATTCTGAACTCGAGTTCAGAATGGATCTTTAAGGCTTTTTTG-3'
shESRP2_2	5'-CCGGCATTGAAGACATCCTGAGCTTCTCGAGAAGCTCAGGATGTCTTCAATGTTTTTG-3'
shRBFOX2_1	5'-CCGGTTGGCGCTGTGGCGAGTTTATCTCGAGATAAACTCGCCACAGCGCCAATTTTTG-3'
shRBFOX2_2	5'-CCGGGTATATGGTCCGGAGTTATATCTCGAGATATAACTCCGGACCATATACTTTTTG-3'
shZEB1	5'-CCGGCGGCGCAATAACGTTACAAATCTCGAGATTTGTAACGTTATTGCGCCGTTTTT-3'
eGFP shControl	5’-CCGGTACAACAGCCACAACGTCTATCTCGAGATAGACGTTGTGGCTGTTGTATTTTT-3’

<u>Supplementary Table 9:</u> Clinical characteristics of patients

Sl.No.	Patient No.	Histopathology	Estrogen(ER), Progesterone(PR), Her2 Status
1	Patient 1	Grade II T2N0Mx	ER(-ve) PR(-ve) Her2(-ve) (TNBC)
2	Patient 2	Grade II T2N1Mx Infiltrating duct carcinoma	ER(-ve) PR(-ve) Her2(+ve)
3	Patient 3	Grade III T2N1Mx Infiltrating duct carcinoma	ER(-ve) PR(-ve) Her2(-ve) (TNBC)
4	Patient 4	Grade III	
5	Patient 5	Grade II T1N0Mx	ER(+ve) PR(+ve) Her2(-ve)
6	Patient 6	Grade II T2N1Mx	ER(+ve) PR(+ve) Her2(-ve)
7	Patient 7	Grade II T1N0Mx	ER(-ve) PR(-ve) Her2(-ve) (TNBC)
8	Patient 8	Grade II T2N1Mx	ER(+ve) PR(weak +ve) Her2(equivocal)
9	Patient 9	Grade I T2N0Mx	ER(weak +ve) PR(-ve) Her2(equivocal)
10	Patient 10	Grade III T3N0Mx	ER(-ve) PR(-ve) Her2(-ve) (TNBC)
11	Patient 11	Malignant Phyllodes tumor	

12	Patient 12	Grade II T3N0Mx	ER(-ve) PR(-ve) Her2(-ve) (TNBC)
13	Patient 13	Grade III T3N0Mx	ER(-ve) PR(-ve) Her2(-ve) (TNBC)
14	Patient 14	Grade III T4N0Mx	ER(-ve) PR(-ve) Her2(weak +ve)
15	Patient 15	ypT2N1Mx	ER(+ve) PR(+ve) Her2(2+ equivocal)
16	Patient 16	T2N2Mx	ER(+ve) PR(+ve) Her2(+ve)
17	Patient 17	Grade II T2N1Mx	ER(+ve) PR(weak +ve) Her2(2+ equivocal)
18	Patient 18	Grade II T2N0Mx	ER(+ve) PR(-ve) Her2(+ve)
19	Patient 19	T2N2Mx Infiltrating duct carcinoma	ER(+ve) PR(-ve) Her2(+ve)

Supplementary Table 10: Primers used in Chromatin immunoprecipitation (ChIP)

S.No	Primers	Sequence
1	SLUG Fw	GCCTGCCTTTAGAGGGCTAC
2	SLUG Rev	TGCGCTACTCAGGGCTTC
3	RBFOX2 Fw	AGGCCTGCTTCTGGAGTTG
4	RBFOX2 Rev	TTCCTCTCTGGCAGTCTCTCC
5	ESRP1 Fw (SULG ChIP)	CTGGCCTTCGCCCGCTCTCA
6	ESRP1 Rev (SULG ChIP)	AGAAGGTGCAGGCGGAGGC
7	ESRP1 Fw (RBFOX2 ChIP)	TTACCGGATTTGTACCCGGA

8	ESRP1 Rev (RBFOX2 ChIP)	ATTCGGCTCTAGGTGTCCAG

Supplementary Figure legends:

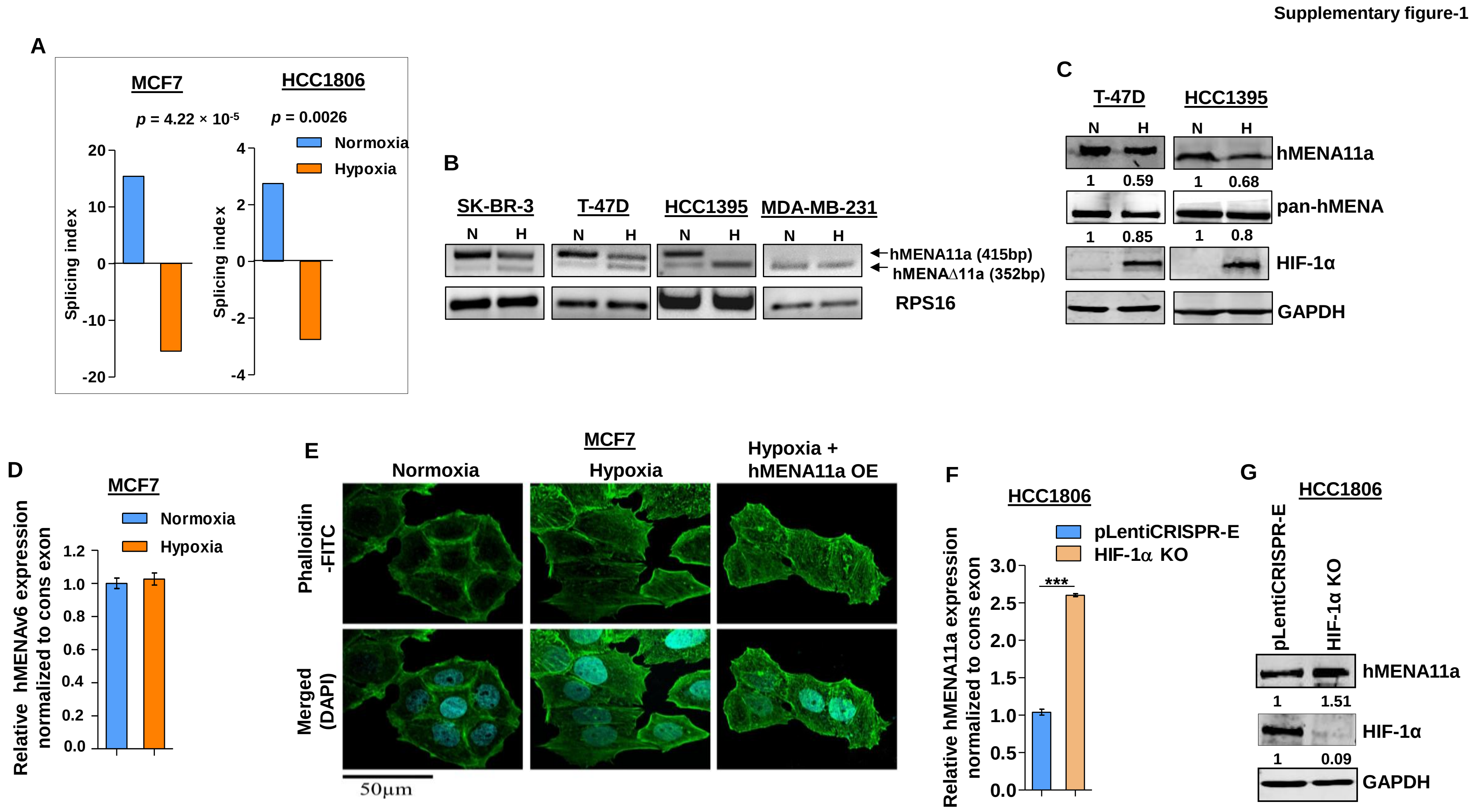

Supplementary Figure S1. hMENA11a isoform is downregulated under hypoxia which affects actin polymerization and invasive properties of breast cancer cells**.** (A) Microarray using Human Transcriptome Array 2.0 was performed to analyze the alternative splicing events post hypoxic treatment in breast cancer cell lines MCF7 and HCC1806. The significant exclusion of exon 11a under hypoxia as compared to normoxia for hMENA gene is represented by splicing index values for MCF7 and HCC1806. (n=2) (B) Semi-quantitative of *hMENA* showing upper (exon 11a included) and lower (exon 11a excluded) bands under normoxia and hypoxia in SKBR3, T47D, HCC1395 and MDAMB231. (C) Immunoblotting of hMENA11a, pan-hMENA and HIF-1α under normoxia and hypoxia in T47D and HCC1395. (D) qRT-PCR of *hMENAv6* under normoxia and hypoxia in MCF7 (Ct values are normalized to RPS16). (E) Phalloidin staining under normoxia, hypoxia and hMENA11a overexpression under hypoxic conditions in MCF7. (F) qRT-PCR for hMENA11a splicing and (G) Immunoblot for hMENA11a and HIF-1α in HIF-1α knockout HCC1806 cells under hypoxia. Error bar shows mean values ± SD. (n=3 unless otherwise specified). As calculated using two-tailed Student's t test, ***$p < 0.001$.

Supplementary figure-2

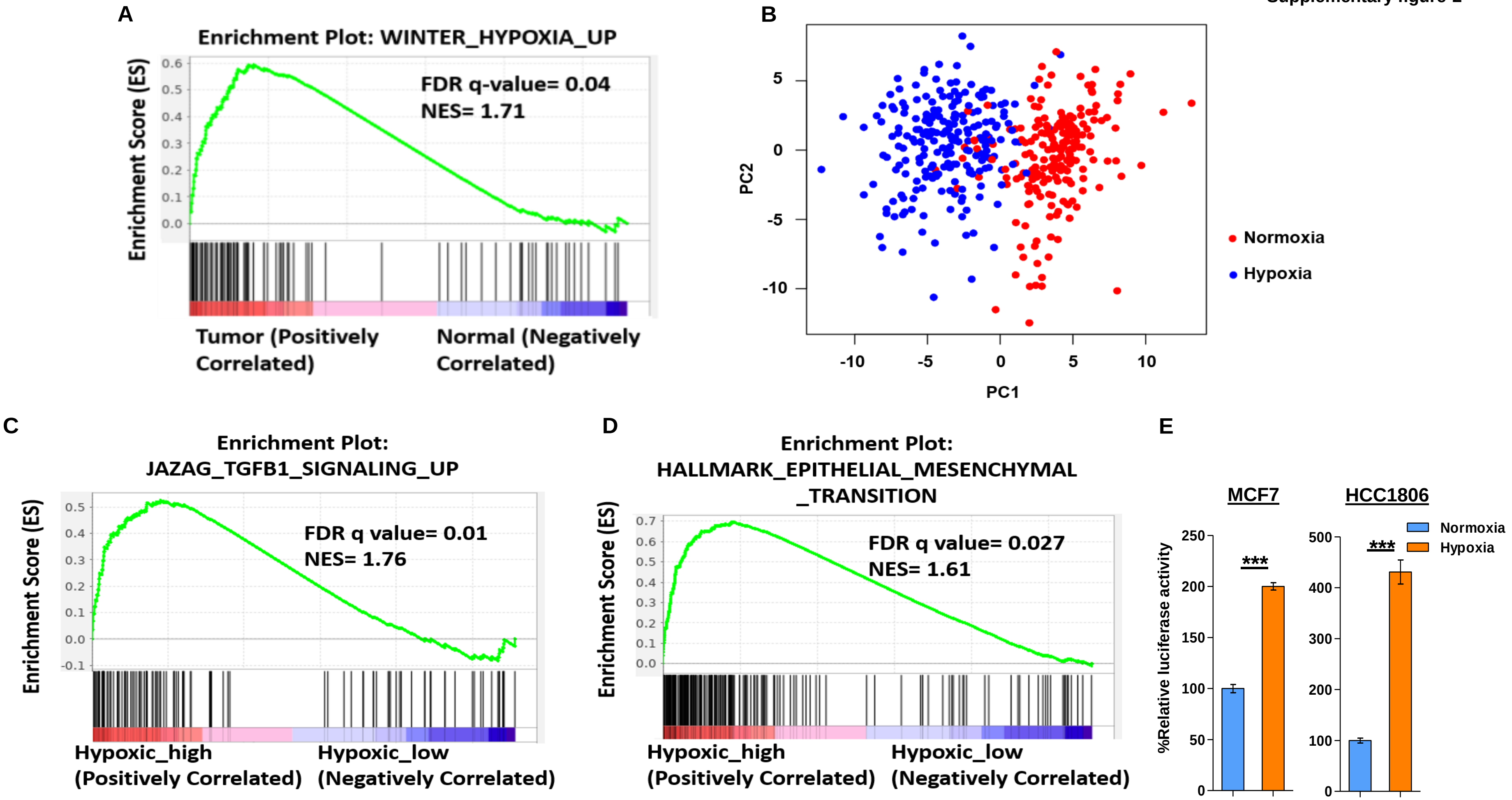

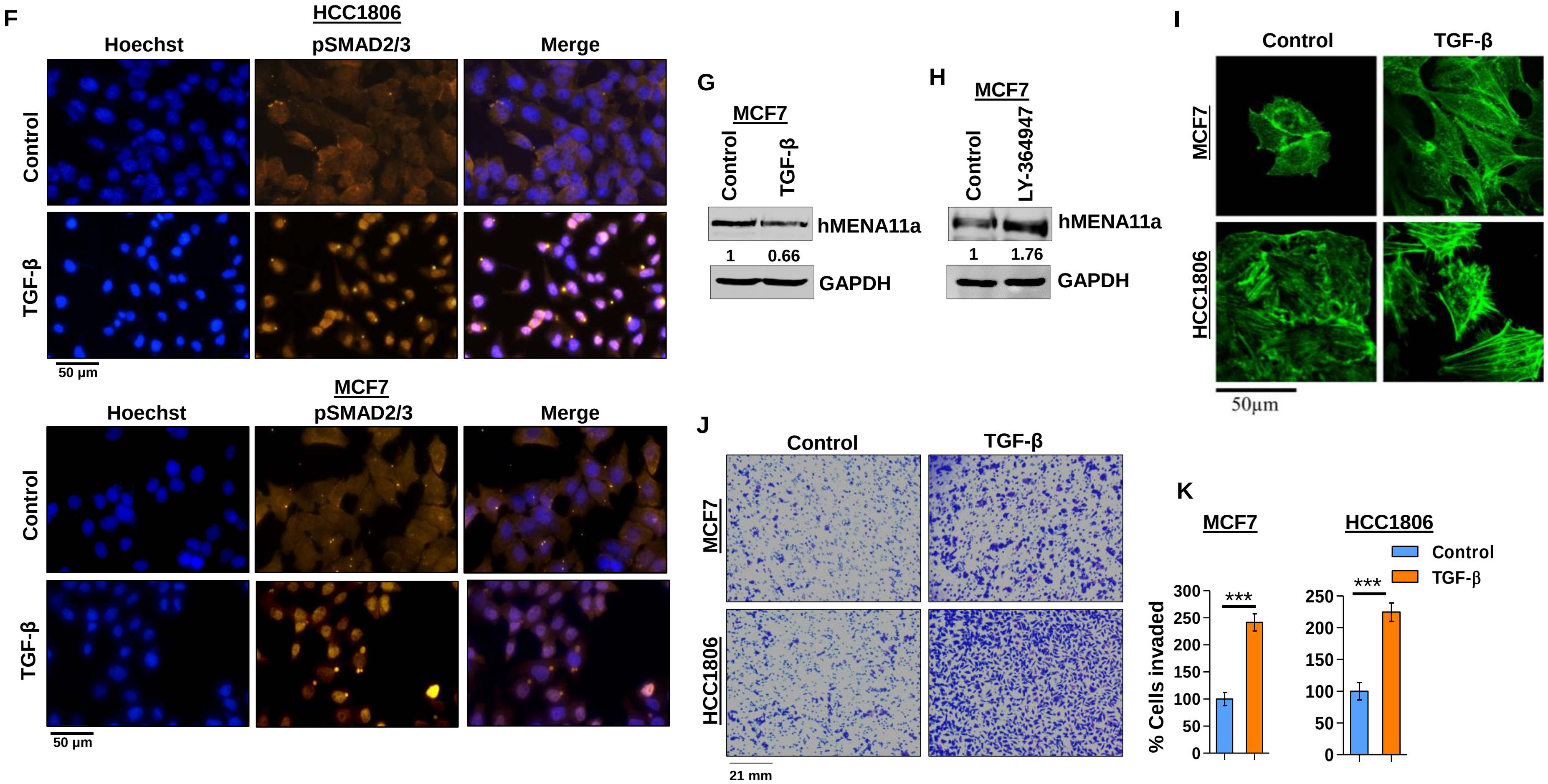

F
HCC1806
Hoechst
pSMAD2/3
Merge
Control
TGF-β
50 μm
MCF7
Hoechst
pSMAD2/3
Merge
Control
TGF-β
50 μm
G
MCF7
Control
TGF-β
hMENA11a
1
0.66
GAPDH
H
MCF7
Control
LY-364947
hMENA11a
1
1.76
GAPDH
I
Control
TGF-β
MCF7
HCC1806
50μm
J
Control
TGF-β
MCF7
HCC1806
21 mm
K
MCF7
HCC1806
Control
TGF-β
% Cells invaded
***
***

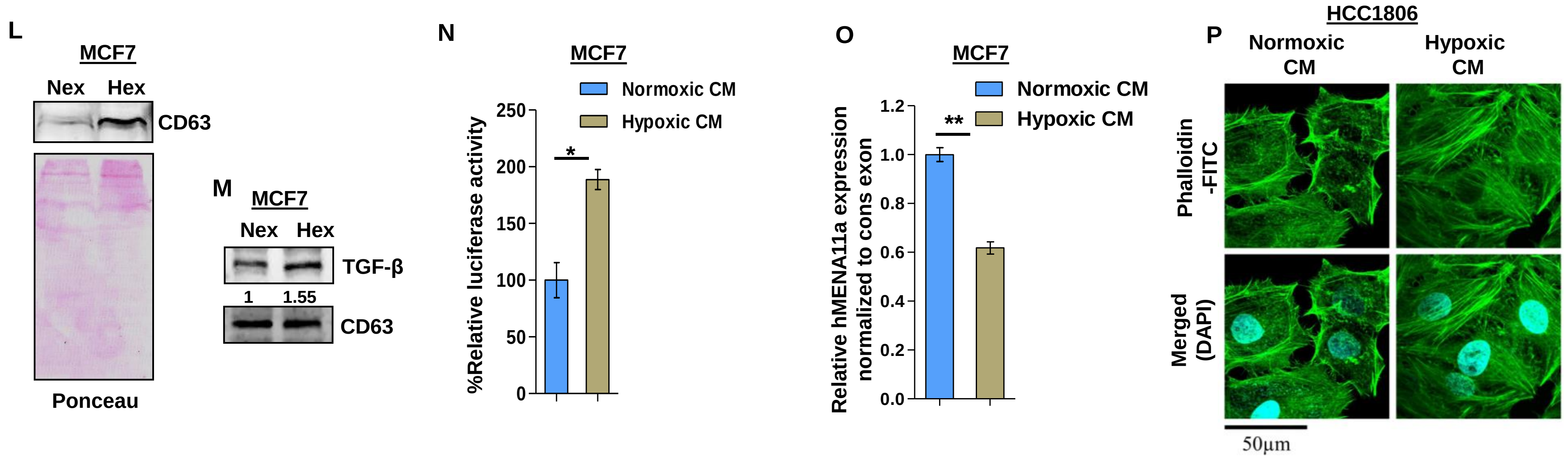

Supplementary Figure S2. Activation of TGF-β signaling under hypoxia causes exon11a exclusion from *hMENA* pre-mRNA. (A) GSEA enrichment plot for hypoxia for breast cancer (n=1099) versus corresponding normal (n=113) samples from TCGA. (B) Scatter plot of principal component 1 (PC1) vs. principal component 2 (PC2) representing the 2 clusters, Hypoxia_high (blue) and Hypoxia_low (red). The plot was generated after performing unsupervised K-means clustering of gene expression data corresponding to Hypoxia_high and Hypoxia_low groups followed by principal component analysis. The partitioning of the 2 clusters shows the confidence of our stratification. (C) GSEA enrichment plot for TGF-β pathway for TCGA breast cancer samples stratified as Hypoxic_high (n=220) or Hypoxic_low (n=220). (D) GSEA enrichment plot for EMT pathway for TCGA breast cancer samples stratified as Hypoxic_high (n=220) or Hypoxic_low (n=220). (NES, Normalized Enrichment Score). (E) Relative luciferase activity normalized to empty vector as well as Renilla after 12 hrs of normoxic versus hypoxic treatment in MCF7 and HCC1806. (F) Immunofluorescence of pSMAD2/3 showing enhanced nuclear localization under TGF-β (10ng/ml) treatment as compared to control in MCF7 and HCC1806 after 12 hrs under normoxia. (G) Immunoblot of hMENA11a after TGF-β (10ng/ml) treatment under normoxia in MCF7. (H) Immunoblot of hMENA11a after TGF-β inhibitor treatment under hypoxia in MCF7. (I) Phalloidin

staining after 24 hrs of TGF-β(10ng/ml) treatment under normoxia in MCF7 and HCC1806. (J) Invasion assay and its (K) quantification and (L) Immunoblot of CD63 exosomal marker showing enhanced expression in hypoxic versus normoxic exosomes. (M) Immunoblot for TGF-β in normoxic and hypoxic exosomes (CD63 exosomal marker was used as control) collected from media of MCF7 treated under normoxia and hypoxia respectively. (N) Relative luciferase activity after 12 hrs of normoxic and hypoxic conditioned media treatment in MCF7. (O) qRT-PCR for *hMENA11a*, 24 hrs after normoxic and hypoxic conditioned media treatment in MCF7. (P) Phalloidin assay 24 hrs after normoxic and hypoxic conditioned media treatment in HCC1806. Error bar shows mean values ± SD. (n=3 unless otherwise indicated). As calculated using two-tailed Student's t test, $*p < 0.05$, $**p < 0.01$, $***p < 0.001$.

Supplementary figure-3

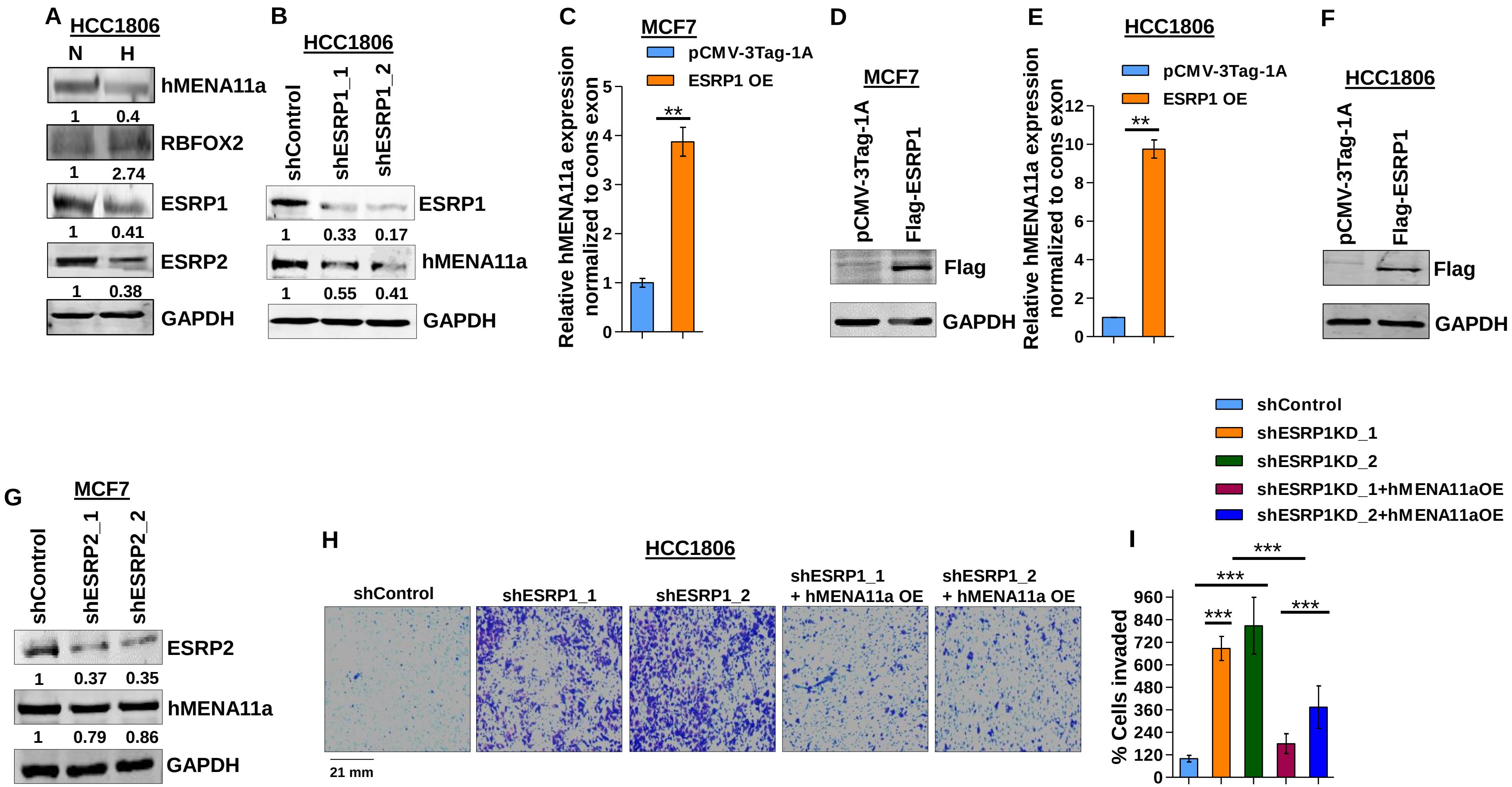

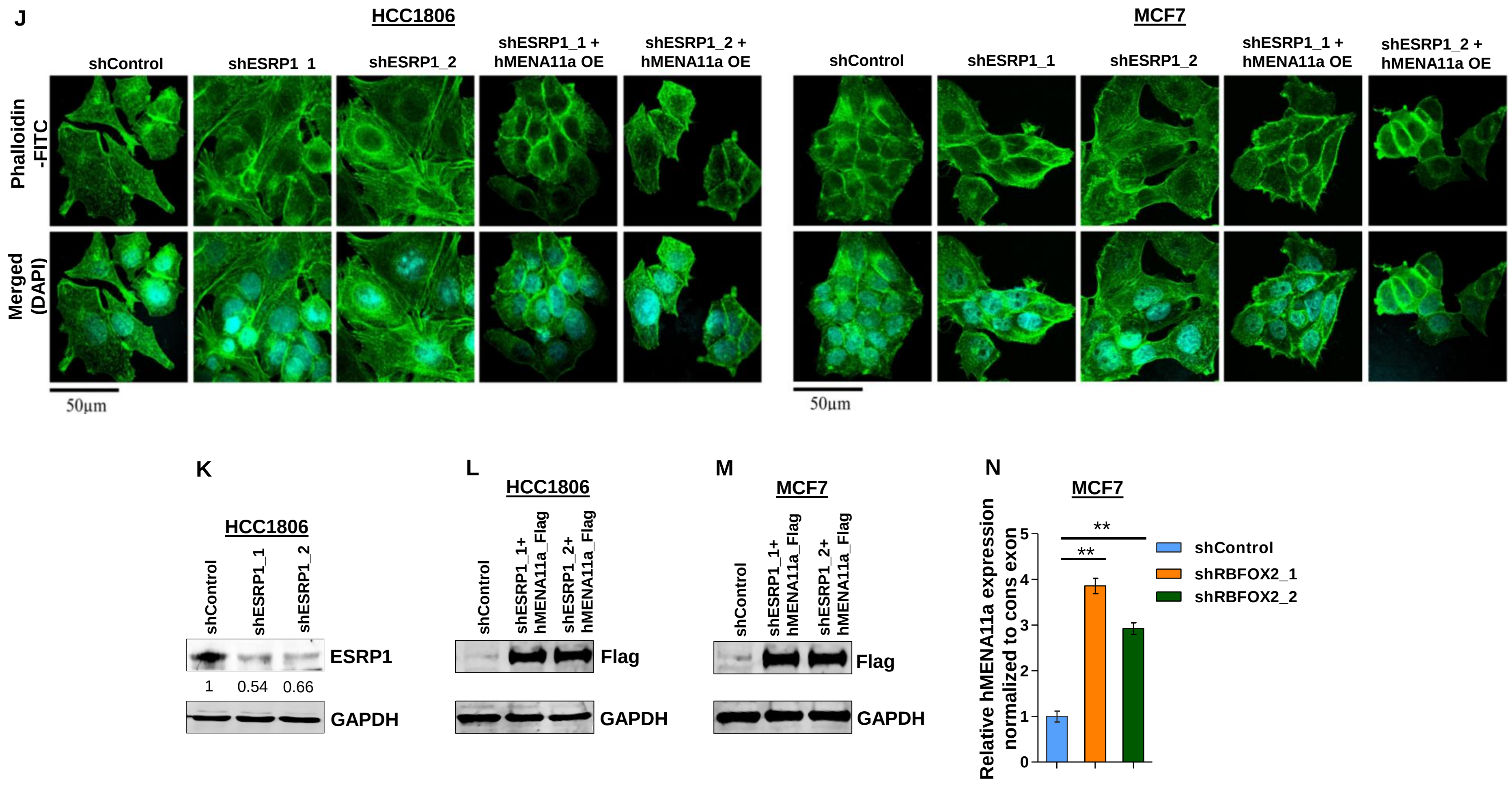

Supplementary Figure S3. ESRP1 is downregulated under hypoxia and leads to exon 11a exclusion from *hMENA* pre-mRNA. (A) Immunoblot of hMENA11a, RBFOX2, ESRP1, ESRP2 under hypoxia versus normoxia in HCC1806. (B) Immunoblot of ESRP1 and hMENA11a after ESRP1 knockdown in HCC1806 under normoxic condition. (C) qRT-PCR for *hMENA11a* after ESRP1 overexpression under hypoxic

conditions in MCF7 and (D) Flag immunoblot for validation of ESRP1 overexpression. (E) qRT-PCR for *hMENA11a* after ESRP1 overexpression under hypoxic conditions in HCC1806 and (F) Flag immunoblot for validation of ESRP1 overexpression. (G) Immunoblot of ESRP2 and hMENA11a after ESRP2 knockdown in MCF7 under normoxic condition. (H) Invasion assay and its (I) quantification as (% of cells invaded) in HCC1806 and (J) Phalloidin staining in MCF7 and HCC1806 after ESRP1 knockdown and rescue with hMENA11a ectopic expression in knockdown cells under normoxia. (K) Immunoblot for ESRP1 in ESRP1 knockdown cells vs control in HCC1806 and immunoblot of Flag to confirm ectopic hMENA11a expression in ESRP1 knockdown (L) HCC1806 and (M) MCF7 cells under normoxia. (N) qRT-PCR for *hMENA11a* after RBFOX2 knockdown under hypoxic condition in MCF7(n=2). Error bar shows mean values ± SD. (n=3 unless otherwise indicated). As calculated using two-tailed Student's t test, **$p < 0.01$, ***$p < 0.001$.

Supplementary figure-4

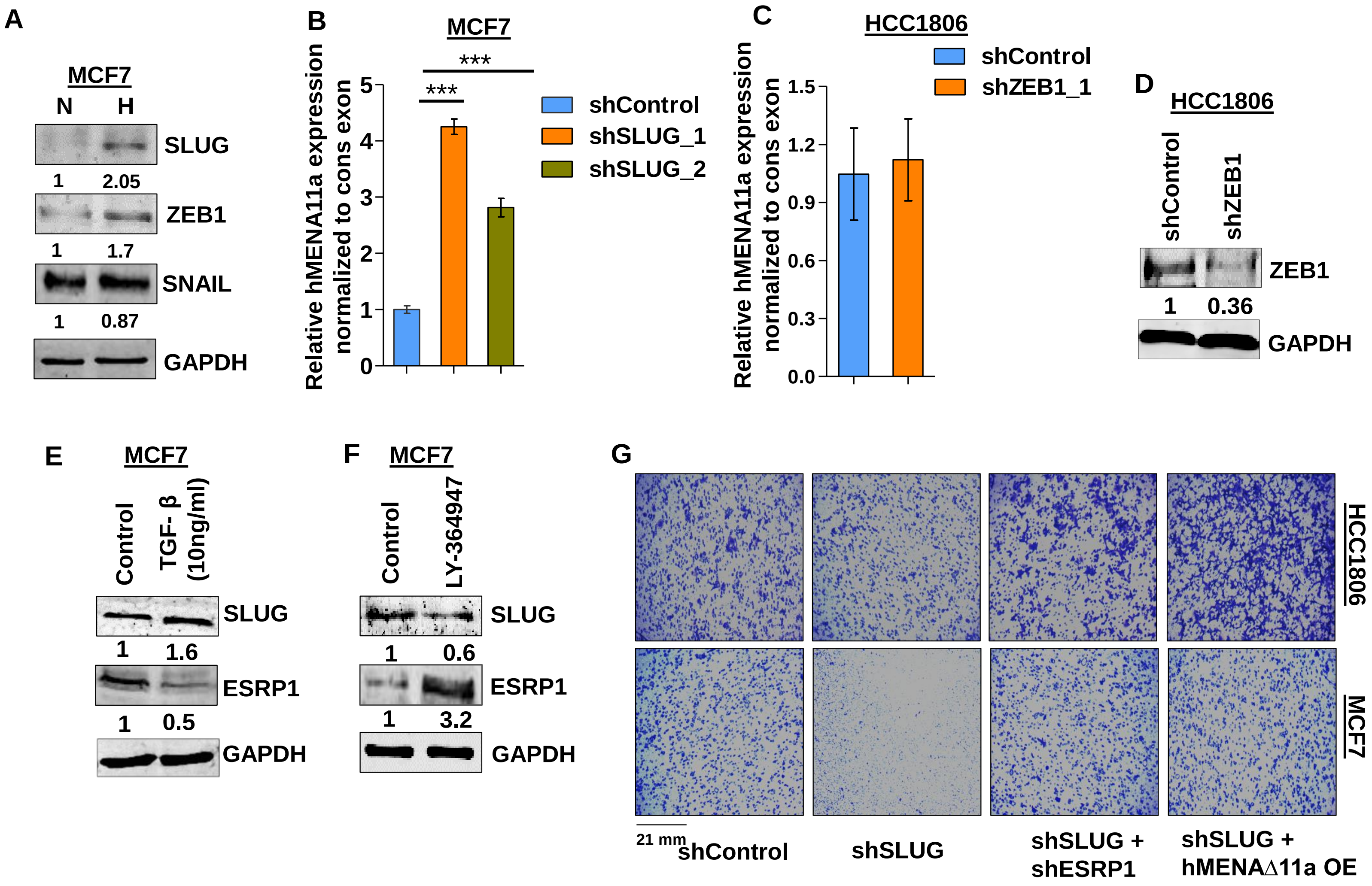

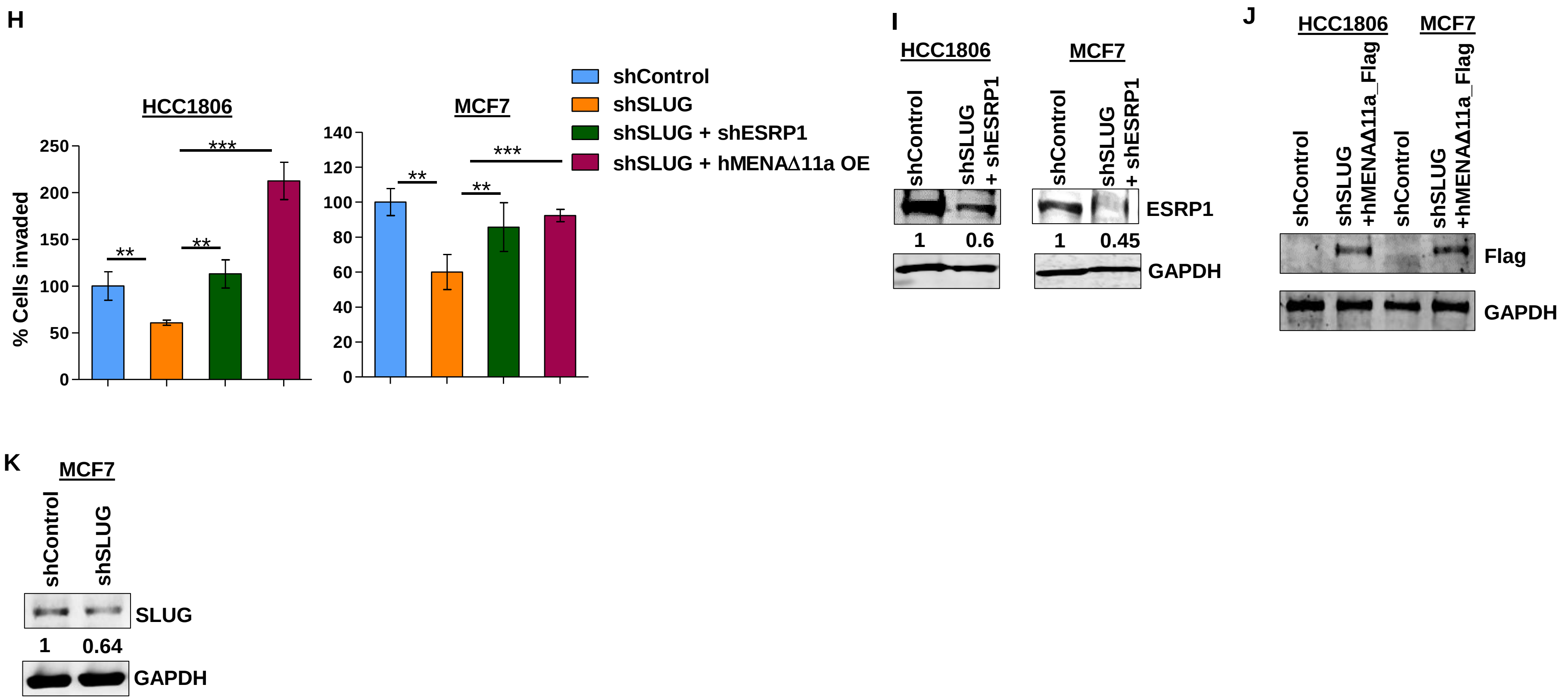

Supplementary Figure S4. SLUG upregulation under hypoxia downregulates ESRP1 and causes exon 11a exclusion from *hMENA* pre-mRNA. (A) Immunoblot of SLUG, ZEB1 and SNAIL in normoxia vs hypoxia in MCF7. (B) qRT-PCR of *hMENA11a* isoform after SLUG knockdown in MCF7 under hypoxic condition. (C) qRT-PCR of *hMENA11a* splicing and (D) Immunoblot of hMENA11a isoform after ZEB1 knockdown in HCC1806 under hypoxic condition. Immunoblot of SLUG, ESRP1 after (E) TGF-β (10ng/ml) and (F) TGF-β inhibitor treatment in MCF7 under

normoxia and hypoxia respectively. (G) Invasion assay with (H) quantification as % of cells invaded after SLUG knockdown, SLUG and ESRP1 double knockdown and hMENAΔ11a ectopic expression in knockdown cells under hypoxia in HCC1806 and MCF7. (I) Immunoblot of ESRP1 in SLUG and ESRP1 double knockdown cells versus control and (J) Immunoblot of Flag to confirm overexpression of hMENAΔ11a isoform in SLUG knockdown HCC1806 and MCF7 cells under hypoxia. (K) Immunoblot of SLUG on SLUG knockdown in MCF7 (GAPDH as control). Error bar shows mean values ± SD. (n=3 unless otherwise indicated). As calculated using two-tailed Student's t test, $**p < 0.01$, $***p < 0.001$.

Supplementary figure-5

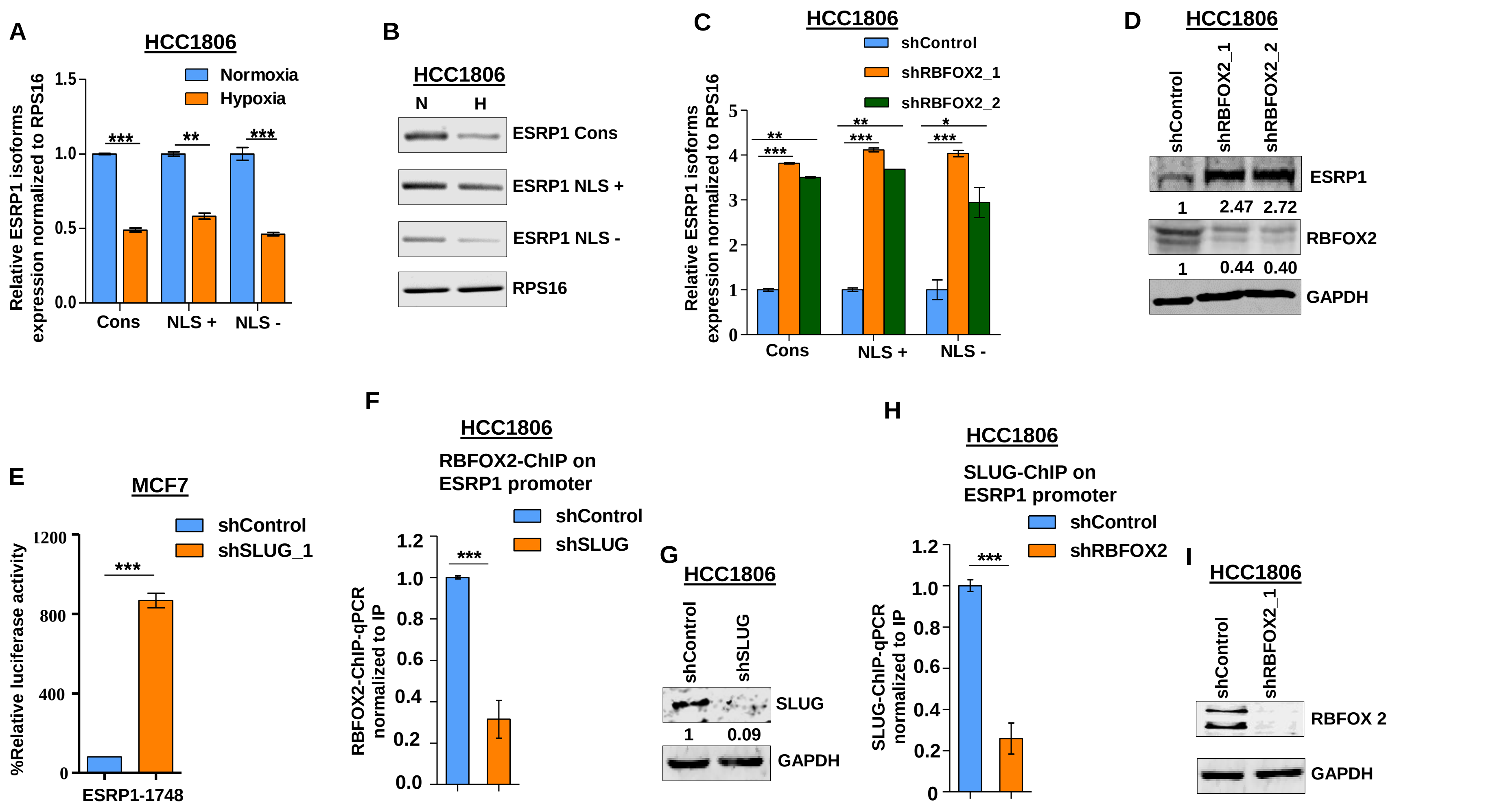

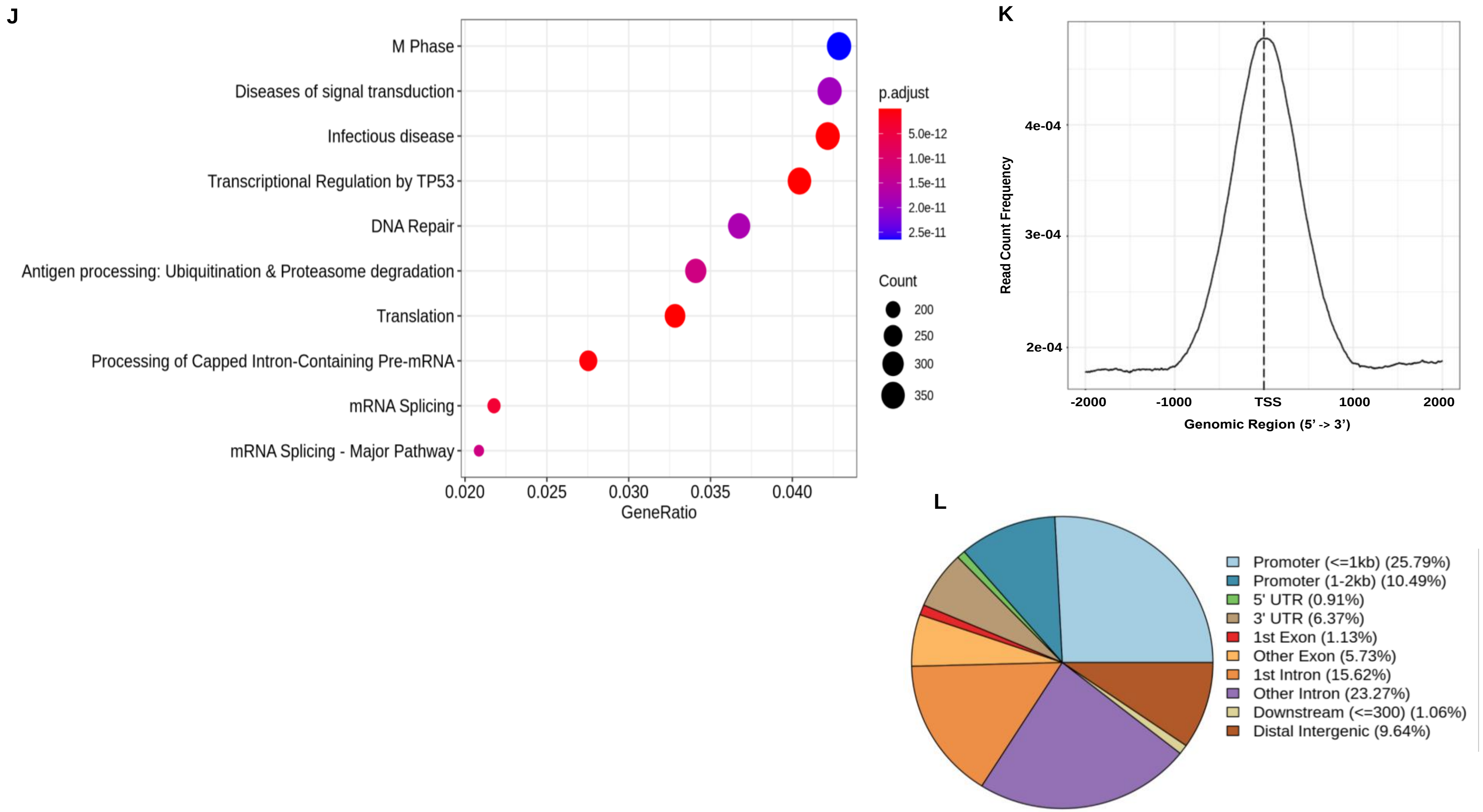

Supplementary Figure S5. Hypoxia-driven TGF-β signaling upregulates RBFOX2, which in turn negatively regulates ESRP1 expression. (A) qRT-PCR and (B) Semi-quantitative PCR to measure the splicing pattern of the *ESRP1* gene under normoxia and hypoxia in HCC1806 cells.

Indicated isoform-specific primers were used and normalized with RPS16. (C) qRT-PCR to measure the splicing isoforms of ESRP1 after RBFOX2 knock-down in HCC1806 cells. Indicated isoform-specific primers were used and normalized with RPS16. (NLS is Nuclear Localization Signal) (D) Immunoblot for ESRP1 and RBFOX2 expression in HCC1806 cells after RBFOX2 knock-down under hypoxia. (GAPDH as control). (E) After the transfection of *SLUG* shRNA or shControl, MCF7 cells were incubated under hypoxia for 24 hrs. Thereafter, *ESRP1* -1748 reporter plasmids were transfected and incubated for 24 hrs, and the reporter assays were carried out. The relative luciferase values are shown as mean ± SD. (F) ChIP qRT-PCR on ESRP1 promoter using RBFOX2 antibody in SLUG knockdown HCC1806 cells. Fold enrichment (RBFOX2/IgG) was normalized to 5% input. (G) Immunoblot to confirm knockdown of SLUG in HCC1806 cells. (H) ChIP qRT-PCR on ESRP1 promoter using SLUG antibody in RBFOX2 knockdown HCC1806 cells. Fold enrichment (SLUG /IgG) was normalized to 5% input. (I) Immunoblot to confirm knockdown of RBFOX2 in HCC1806 cells. (J) Enriched Reactome Pathway terms obtained after performing Over Representation Analysis on genes with RBFOX2 binding around TSS. (K) Occupancy profile of RBFOX2 around 2 kilo base pairs of transcription start sites (TSS) in dataset GSE106042. (L) Pie chart showing the distribution of RBFOX2 ChIP-seq peaks across several genomic features. Error bar shows mean values ± SD. (n=3 unless otherwise indicated). As calculated using two-tailed Student's t test, $*p < 0.05$, $**p < 0.01$, $***p < 0.001$.

Supplementary figure-6

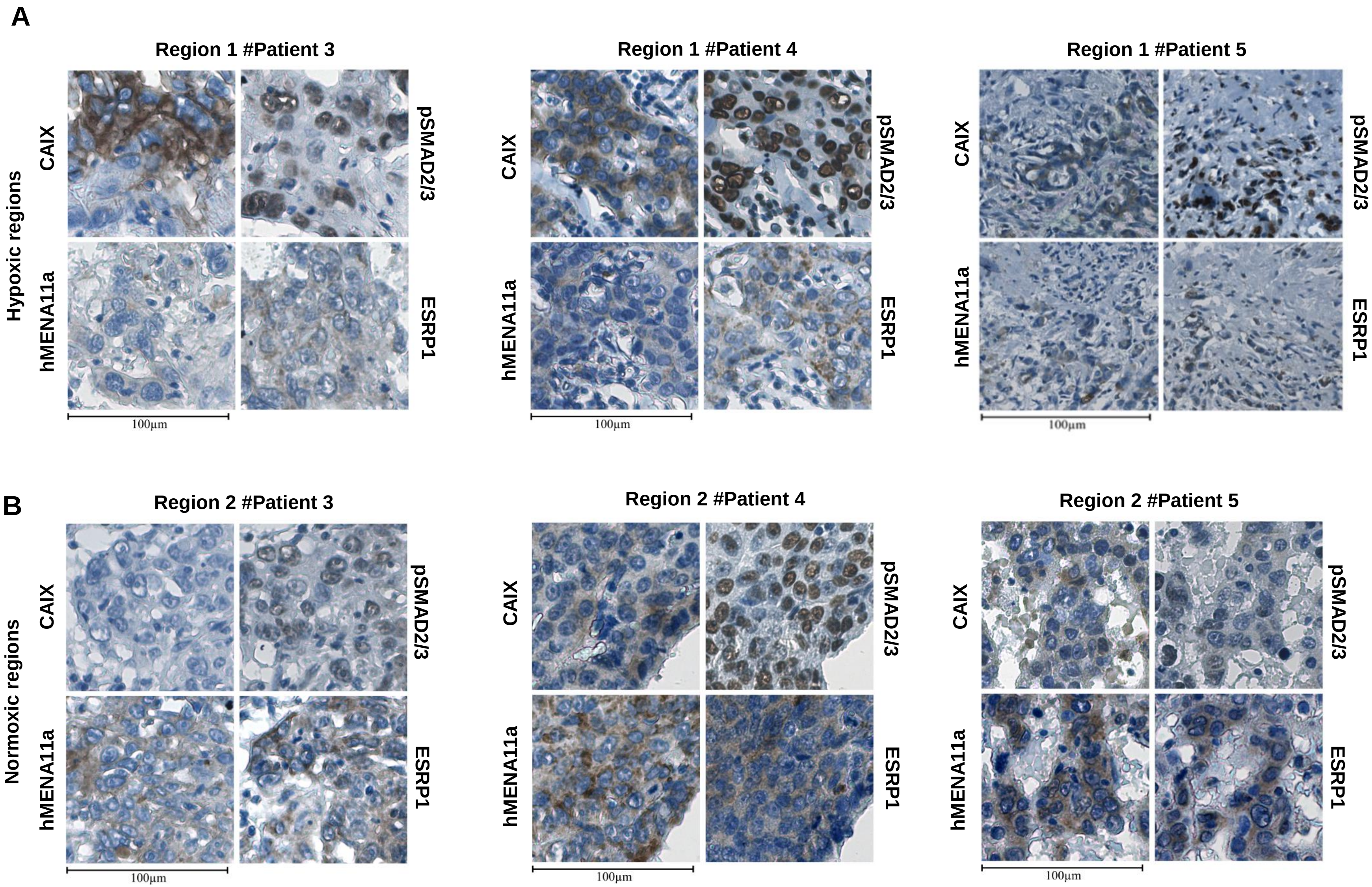

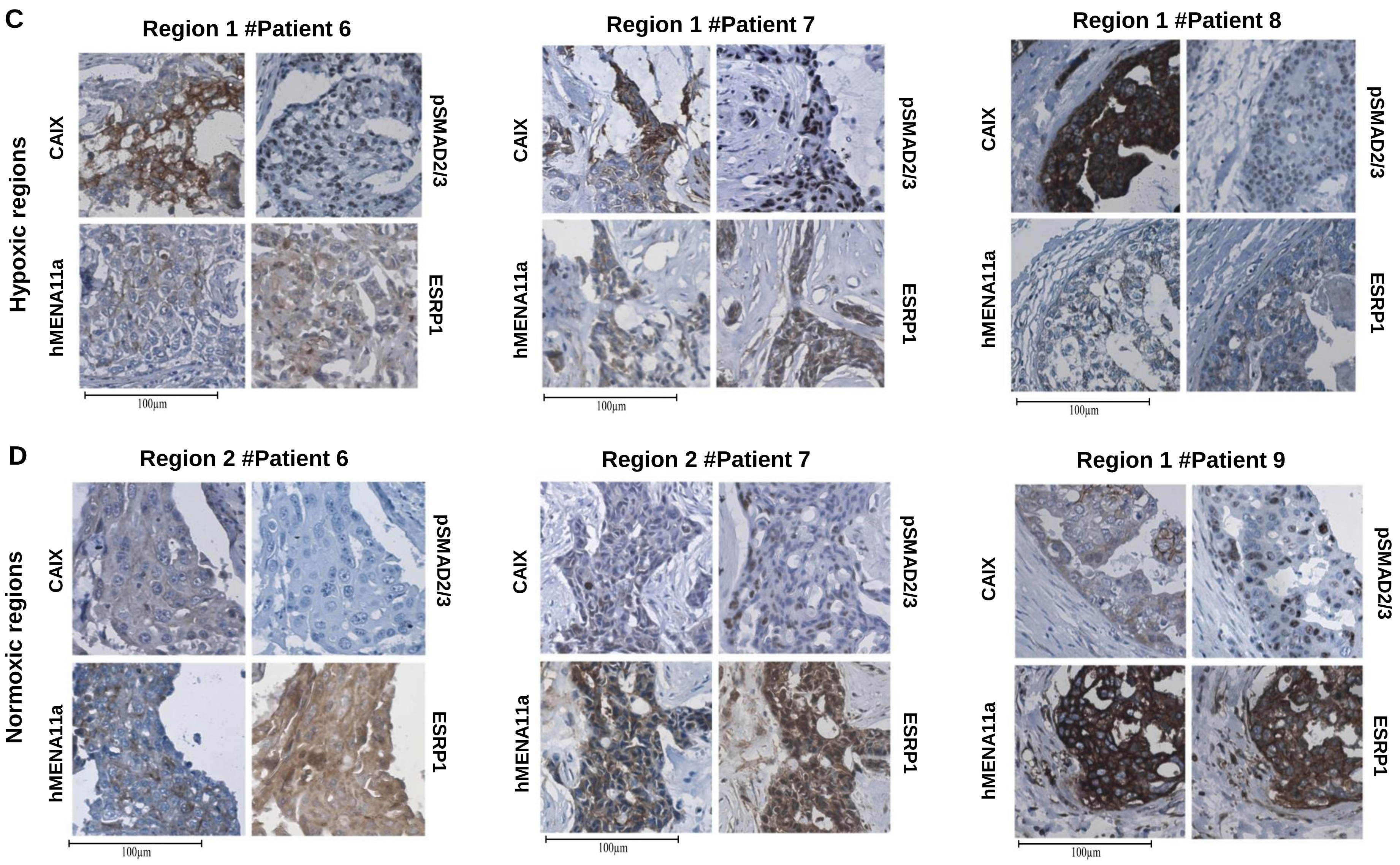
C
Region 1 #Patient 6
Region 1 #Patient 7
Region 1 #Patient 8
Hypoxic regions
CAIX
pSMAD2/3
hMENA11a
ESRP1
100µm
D
Region 2 #Patient 6
Region 2 #Patient 7
Region 1 #Patient 9
Normoxic regions
CAIX
pSMAD2/3
hMENA11a
ESRP1
100µm

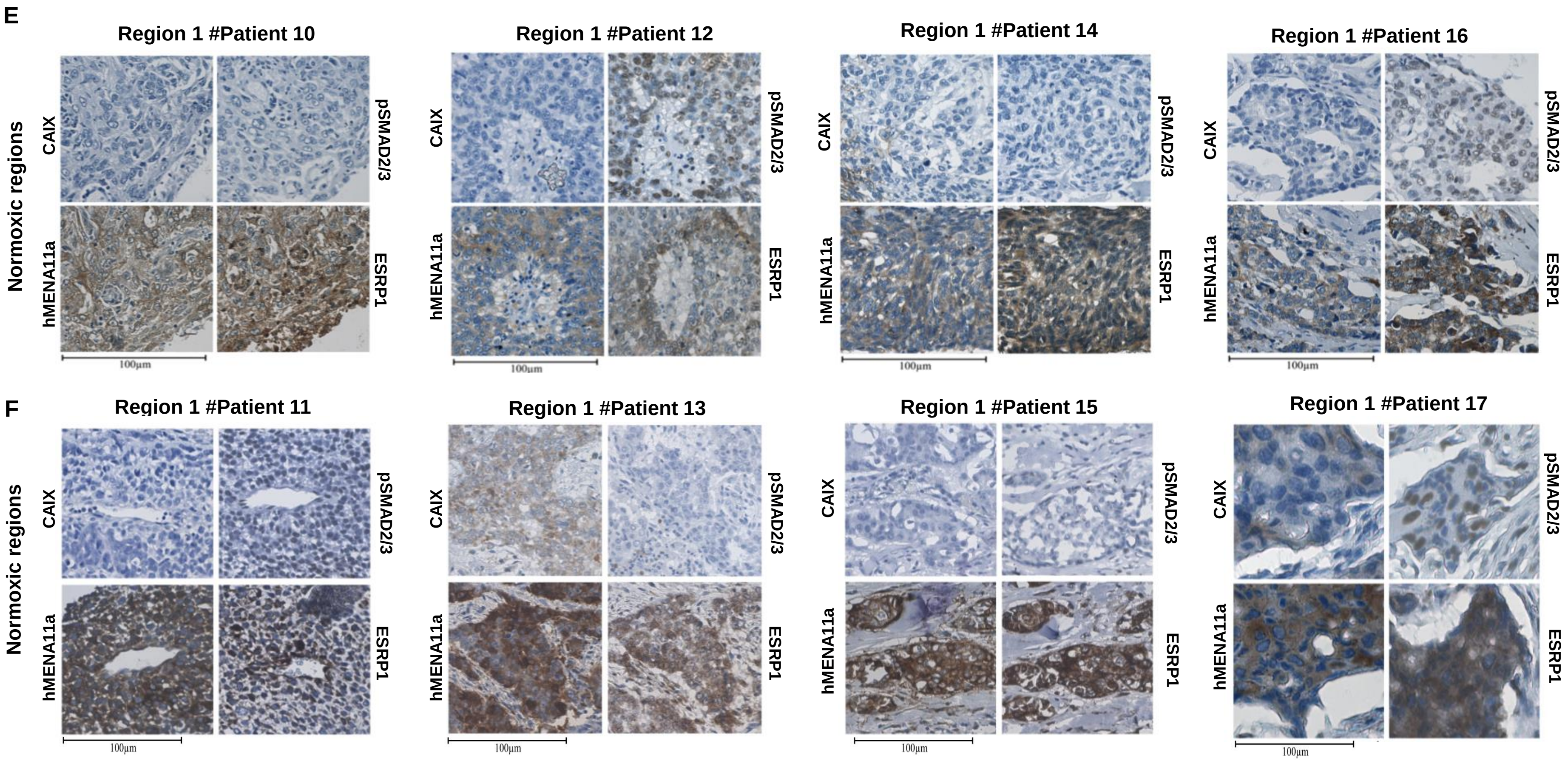
E
Region 1 #Patient 10
Region 1 #Patient 12
Region 1 #Patient 14
Region 1 #Patient 16
Normoxic regions
CAIX
pSMAD2/3
hMENA11a
ESRP1
100µm
F
Region 1 #Patient 11
Region 1 #Patient 13
Region 1 #Patient 15
Region 1 #Patient 17
Normoxic regions
CAIX
pSMAD2/3
hMENA11a
ESRP1
100µm

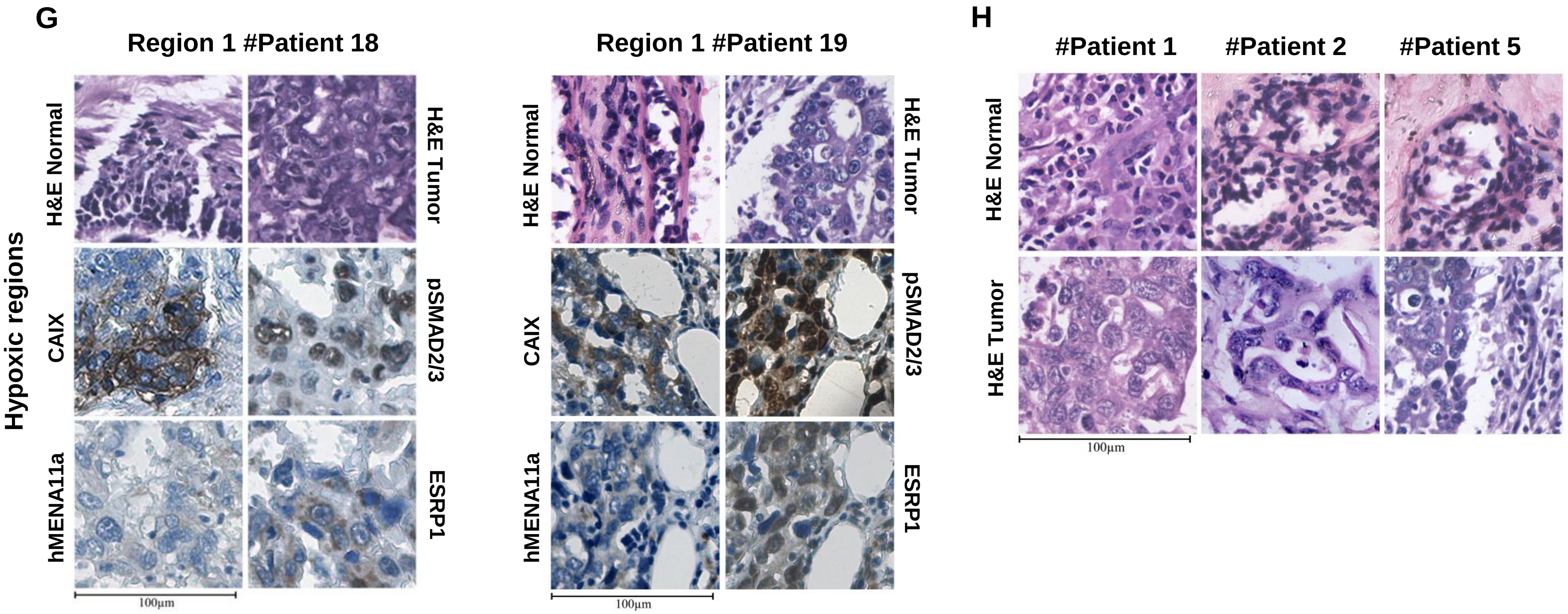

Supplementary Figure S6. CA IX, pSMAD2/3, hMENA11a, ESRP1 immunostaining of seventeen illustrative cases of breast cancer patients. (A, C and G) Hypoxic regions: Areas showing strong membranous and/or cytoplasmic immunostaining for CA IX also exhibit strong expression of pSMAD2/3 (nuclear) and weak expression of hMENA11a and ESRP1 (cytoplasmic). (B, D, E and F **)** Normoxic regions: Areas showing weak immunostaining for CA IX also exhibit weak expression of pSMAD2/3 and strong expression of both hMENA11a and ESRP1, are shown for (A and B) Patient 3, 4, and 5, (C and D) Patient 6, 7, 8 and 9 (E, F and G) Patient 10-19. (H) Hematoxylin and eosin staining for Patients 1, 2, and 5. Magnification, 40x. Error bar shows mean values ± SD. (n=3 unless otherwise indicated). As calculated using two-tailed Student's t test, $^{**}p < 0.01$, $^{***}p < 0.001$.

Supplementary figure-7

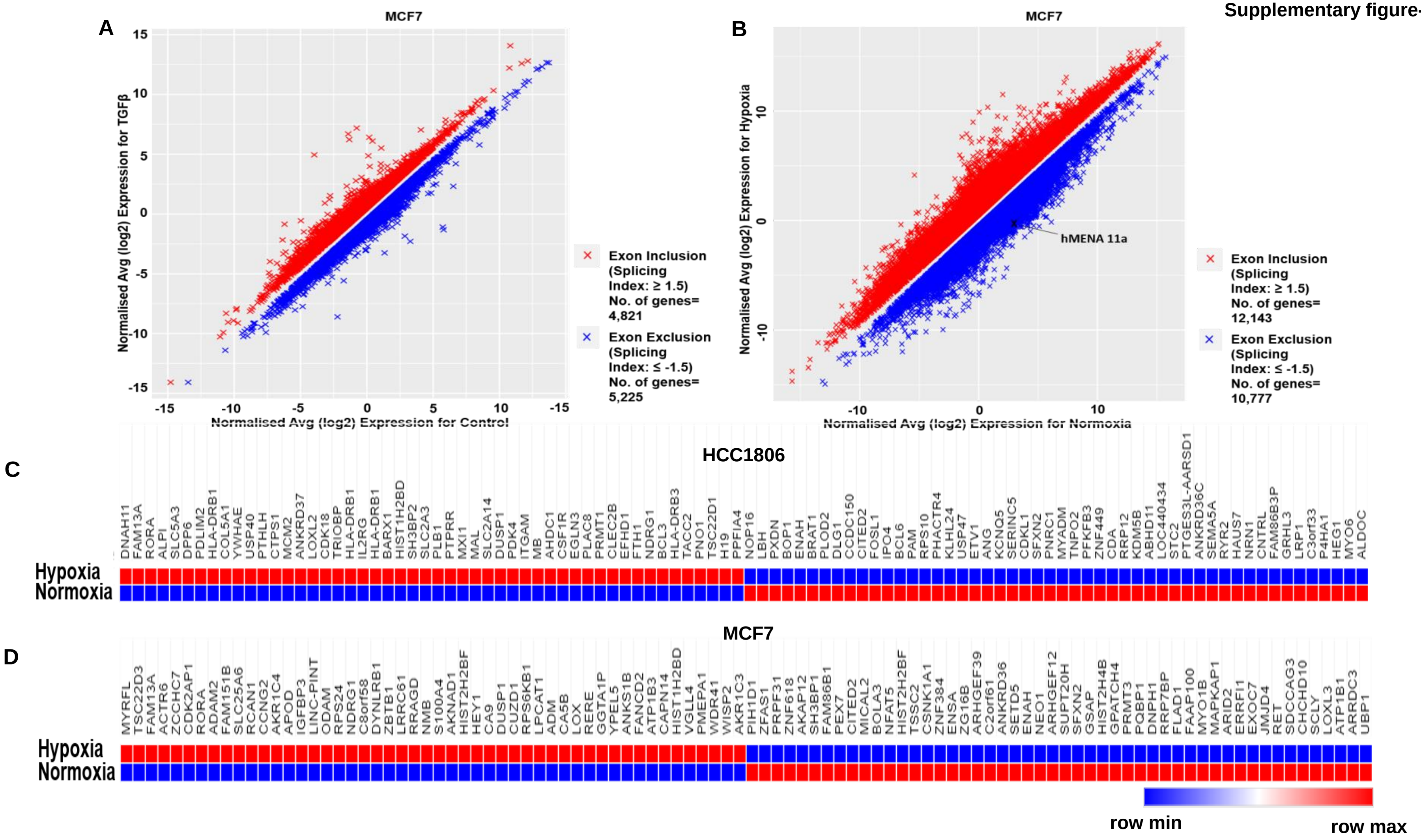

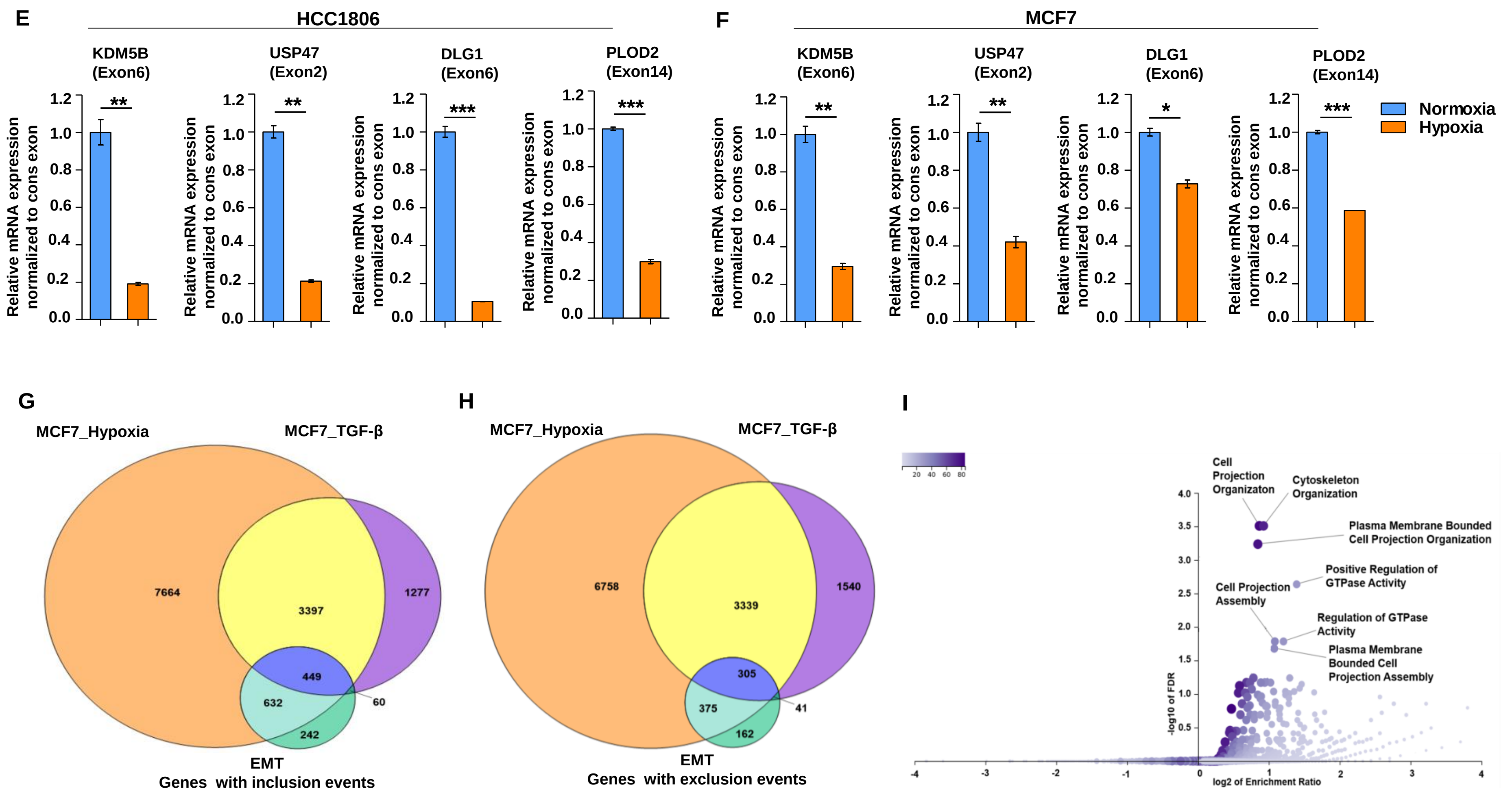

Supplementary Figure S7. Global effect of TGF-β signaling on alternative splicing under hypoxia in breast cancer cell line MCF7. (A) Microarray HTA 2.0 profile showing differential splicing events in TGF-β treated versus control MCF7 cells (n=2, $|SI| \geq 1.5$, $p < 0.05$). (B) Microarray HTA 2.0 profile showing the differential splicing events in hypoxia-treated (cells cultured in 1% O2) versus control (normoxic)

MCF7 cells (n=2, |SI| ≥ 1.5, $p < 0.05$). (C and D) Heatmap showing top 50 inclusion and exclusion cassette exon events observed in HTA-2.0 analysis for HCC1806 and MCF7 between hypoxia and normoxia. (E and F) qRT-PCR for splicing validation of cassette exon exclusion events of genes KDM5B, USP47, DLG1 and PLOD2 under normoxia vs hypoxia in HCC1806 (E) and MCF7 (F). (G) Common genes that show significant exon inclusion events on TGF-β treatment, hypoxia induction and during EMT. 3,846 genes show inclusion under both hypoxia and TGF-β induction while 449 genes show inclusion in all 3 conditions in MCF7. (H) Common genes that show significant exon exclusion events on TGF-β treatment, hypoxia induction and during EMT in MCF7. 3,644 genes commonly show exon exclusion on treatment with hypoxia and TGF-β, whereas 305 genes show exon exclusion in all 3 conditions. (I) Volcano plot representing the enrichment ratio of various Gene Ontology terms over-represented in the set of genes (n= 629) that either show exon inclusion or exclusion in all 3 conditions (FDR < 0.05) (|SI|, absolute splicing index). Error bar shows mean values ± SD. (n=3 unless otherwise indicated). As calculated using two-tailed Student's t test, *$p < 0.05$, **$p < 0.01$, ***$p < 0.001$.
